# Carbon Fiber- vs. Glass Fiber-Reinforced PA12 Under Protic Ionic Liquid Lubrication: Friction, Wear and the Limits of Reinforcement in FFF-Printed Composites

**DOI:** 10.3390/polym18182239

**Published:** 2026-09-14

**Authors:** Horea Stefan Goia, Florin Popister, Corina Birleanu, Razvan Udroiu, Friedemann Schaber, Marius Pustan

**Affiliations:** 1 MicroNano Systems Laboratory, Mechanical Systems Engineering Department, EUt+—Institute for Nanoscience and Nanotechnology of the European University of Technology (EUTINN), Technical University of Cluj-Napoca, Bvd. Muncii No. 103–105, 400641 Cluj-Napoca, Romania; horea.goia@campus.utcluj.ro (H.S.G.); marius.pustan@omt.utcluj.ro (M.P.); 2 Manufacturing Engineering Department, Transilvania University of Brasov, Blv. Eroilor Nr. 29, 500036 Brașov, Romania; udroiu.r@unitbv.ro; 3 Faculty of Arts, Science and Technology, University of Northampton, Northampton NN1 5PH, UK; friedemann.schaber@northampton.ac.uk

**Keywords:** 3D printing, tribology, polyamide 12, composite material, protic ionic liquid, coefficient of friction, specific wear rate, lubrication, reinforced polymers

## Abstract

Fused filament fabrication (FFF) is increasingly employed for functional components subjected to sliding contact, for which friction and wear determine service life as decisively as static strength. Tribological characterization of fiber-reinforced polyamide 12 has been conducted predominantly under dry sliding, and the influence of the lubrication regime on the contrast between reinforcement types remains insufficiently established. The present study investigated carbon fiber (PA12-CF) and glass fiber (PA12-GF) reinforced polyamide 12, both processed by FFF, under dry sliding and under two protic ionic liquid lubricants, bis (2-hydroxyethyl) ammonium succinate (MSu) and bis (2-hydroxyethyl) ammonium citrate (MCi). Discs were tested against a 100Cr6 bearing steel counterface on an Anton Paar TRB3 tribometer at a fixed normal load of 20 N, with sliding speeds of 0.5, 0.75 and 1.0 m/s for 60 min each. The coefficient of friction (COF) and the contact-point temperature were recorded throughout, the specific wear rate was obtained by contact profilometry, and worn surfaces were examined by scanning electron microscopy with energy dispersive X-ray analysis. MCi held the mean COF at 0.023 for both composites, against a dry mean of 0.071 across the study, whereas MSu increased the mean to 0.25 and produced the highest wear rates, concurrent with an increase in viscosity during testing. Reinforcement type governed the morphology of the worn surface consistently, yet lubricant selection proved more consequential than material selection.

## 1. Introduction

### 1.1. FFF for Functional Sliding-Contact Components

Fused filament fabrication (FFF) has evolved from a rapid-prototyping technology into a viable route for manufacturing functional end-use components, including gears, bushings, guides, and other mechatronic parts subjected to sliding contact during service [1,2]. Filaments reinforced with short fibers have been developed alongside this transition, intended to compensate for the low and anisotropic mechanical properties of unreinforced thermoplastics [1]. Their static tensile, flexural, and thermal performance is now well documented. The tribological response of the same materials has attracted considerably less systematic attention, even though friction, wear, and surface damage determine the service life of a sliding component as decisively as static strength does [3,4]. Lubricated sliding remains the least explored case of all, and the scarcity of data extends even to materials as widely used in FFF as acrylonitrile butadiene styrene (ABS), polycarbonate (PC), and polylactic acid (PLA) [5].

### 1.2. PA12 as a Tribological Matrix: Carbon Fiber vs. Glass Fiber Reinforcement

Among the engineering thermoplastics available in filament form, polyamide 12 (PA12) combines moderate hardness with sliding behavior and is well suited to loaded contact. Its lower degree of crystallinity relative to polyamide 6 (PA6) confers greater dimensional stability and a markedly reduced moisture uptake, which matters for a polymer family that is hygroscopic throughout [6]. Reinforcement of FFF-grade PA12 follows two established routes, carbon fiber (PA12-CF) and glass fiber (PA12-GF), and the resulting composites behave differently once sliding begins. Carbon fibers shear at the contact interface and deposit a low-shear-strength, graphitic transfer film onto the metallic counterface, decoupling direct asperity contact and thereby reducing the coefficient of friction while protecting the composite from severe wear [7]. Glass fibers are harder and more brittle. Under contact stress, they fracture, exposing sharp fiber ends and generating pull-out together with matrix fragmentation, which raises the surface hardness of the composite while increasing wear of the counterface [8,9,10]. The two reinforcements therefore appear complementary rather than interchangeable, which motivates a paired comparison under identical sliding conditions.

### 1.3. Nanolubricants and Ionic Liquid Lubricants in Tribological Applications

Lubricant research has increasingly turned to ionic liquids (ILs), which offer negligible vapor pressure, a wide liquid range, and physicochemical properties that can be adjusted through the choice of cation and anion. Within this class, protic ionic liquids (PILs) are obtained by a simple acid–base neutralization between an amine and a carboxylic acid, yielding halogen-free and biodegradable compounds whose only by-product is water [11,12]. Bis (2-hydroxyethyl) ammonium cations paired with short-chain carboxylate anions, among them succinate and citrate, reduce both friction and wear when applied neat and when added to water, with reported friction coefficients between 0.02 and 0.09 and reductions of up to 88% relative to water alone [13]. Bis (2-hydroxyethyl) ammonium succinate (MSu) combines this behavior with a low viscosity of about 0.09 Pa·s at 25 °C [13], while the corresponding citrate salts perform comparably in metal contact [14]. MSu and MCi belong to a family of protic ionic liquids prepared by direct acid–base neutralization of 2-hydroxyethylamine with the corresponding carboxylic acid (succinic or citric acid) [13,14]. Their role is not restricted to neat lubrication. Dispersing nanomaterials such as graphene within a PIL matrix yields nanolubricants that lower friction and wear further and limit surface damage relative to the base fluid alone [15,16]. Tunable chemistry, biodegradability and documented tribological performance together motivate the use of PIL-based lubricants as an alternative to conventional mineral oils in the present study.

### 1.4. Critical Review of Prior Tribological Studies (FFF/SLS, Dry and Lubricated)

Carbon fiber- and glass fiber-reinforced polyamide 12 have each been examined tribologically, but no study identified in the reviewed literature examines them side by side. Gadelmoula and Aldahash characterized the friction and wear behavior of carbon-fiber-reinforced PA12 manufactured by selective laser sintering (SLS) under dry sliding, reporting a 25% reduction in coefficient of friction and 15 to 40% higher wear resistance relative to unreinforced PA12 [8]. The same authors separately investigated glass bead–filled PA12 processed by SLS, again under dry sliding and again without comparison to a fiber-reinforced counterpart [17]. Within the reviewed literature, these SLS-based studies represent the closest available evidence on the tribology of PA12-CF and PA12-GF as material systems. SLS nevertheless produces a powder-sintered and comparatively isotropic microstructure, distinct from the layer-by-layer deposition, weaker interlayer bonding and print-orientation-dependent anisotropy characteristic of FFF, so the findings cannot be assumed to transfer directly to printed parts.

The wider polyamide literature confirms the same divergence in mechanism. Zhang et al. documented the wear and thermal behavior of 3D-printed nylon gears under dry sliding [3], while Kunishima et al. compared carbon fiber- and glass fiber-reinforced polyamide 66 (PA66) against a steel counterface with and without grease lubrication, finding that reinforcement type strongly influenced both composite wear and, unexpectedly, the degree of damage inflicted on the steel counterface itself [10]. Among the studies identified on fiber-reinforced FFF materials, lubricated sliding has been addressed only for one reinforcement type at a time. Luo et al. tested carbon fiber-reinforced FFF-printed nylon under both dry and water lubricated conditions [4], while He et al. modeled the tribological response of unreinforced FFF polymers, including ABS, PC and PLA, across several lubrication modes using a setup methodologically close to the present study [5]. Birleanu et al. characterized glass fiber-reinforced polymer composites under dry conditions against a chrome alloy steel counterface comparable to the one used here [9]. Taken together, these studies show that the two reinforcements produce distinct tribological signatures and that lubrication can substantially alter the friction and wear response of fiber-reinforced polymers. None of those identified in the reviewed literature, however, combines both reinforcement types with more than one lubrication condition within a single controlled protocol for FFF-printed PA12.

### 1.5. Research Gap and Novelty Statement

The reviewed literature reveals a clear gap. Tribological characterization of PA12-CF and PA12-GF exists, but only in isolation, across different manufacturing processes and against different counterface materials, and not under a shared lubrication protocol [8,17]. Work that compares the two reinforcements directly while also examining lubrication appears confined to conventional polyamide composites produced by injection molding. Even there, the outcome is not straightforward, since reinforcement type interacts with interfacial adhesion in ways that fiber hardness alone does not predict [10]. No study identified here combines carbon fiber- and glass fiber-reinforced PA12, produced by fused filament fabrication and tested under both dry and lubricated sliding, within one controlled experimental protocol.

The present study addresses this gap on two fronts. It provides a paired tribological comparison of FFF-printed PA12-CF and PA12-GF under a shared testing protocol, whereas the studies identified treat each reinforcement separately. It also examines how a given lubrication regime modifies the friction and wear contrast between the two rather than treating lubrication and reinforcement as independent variables, which is the approach found in the identified FFF literature.

### 1.6. Research Objectives and Paper Structure

The present study characterizes and compares the coefficient of friction and the wear rate of fused-filament fabricated PA12-CF and PA12-GF discs sliding against a 100Cr6 steel counterface, under dry conditions and under two protic ionic liquid lubricants. Wear rate is obtained by profilometric measurement of the resulting tracks. The influence of sliding speed is evaluated through a configuration in which concentric tracks of differing radius are placed on a single disc. Friction and wear behavior is then related to the surface damage mechanisms operating in each composite, drawing on scanning electron microscopy and energy dispersive X-ray analysis of representative worn surfaces. Section 2 describes the materials and methods, Section 3 presents the results, Section 4 discusses the tribological mechanisms considering the reviewed literature, and Section 5 sets out the conclusions.

## 2. Materials and Methods

### 2.1. Materials

Two commercially available fused-filament fabrication grade polyamide 12 filaments were used, both with a nominal diameter of 1.75 mm. The carbon fiber grade, PA12-CF, carries 20 wt% short fiber, while the glass fiber grade, PA12-GF, carries 15 wt%. Reinforcement type, reinforcement content and processing temperature (Section 2.2) therefore vary together between the two materials, so the comparison reported here reflects their combined effect rather than that of fiber type alone. Section 4 accordingly refers to this combined difference as ‘material’ except where a claim is grounded directly in fiber-composition evidence, for which ‘reinforcement type’ is used. Spools were new and were unsealed immediately before use. Drying followed in a dedicated filament drying box at the manufacturer-recommended settings, a step required by the hygroscopic nature of polyamide 12. Key mechanical and thermal properties, taken from the respective manufacturer technical data sheets, are summarized in Table 1 [18,19].

### 2.2. Specimen Fabrication (FFF)

Disc specimens measuring 32 mm in diameter and 4 mm in thickness were fabricated by fused filament fabrication on an Ender 5 Max printer fitted with the manufacturer’s original equipment enclosure, using a 0.6 mm hardened steel nozzle to accommodate the abrasive fiber-filled filaments. Nozzle and bed temperatures were set to 280 °C and 80 °C for PA12-CF and to 270 °C and 100 °C for PA12-GF. The remaining parameters were common to both materials: a print speed of approximately 40 mm/s, a layer height of 0.3 mm, 20% infill density, six perimeter wall loops and six solid layers at the top and bottom. Each disc was built flat on the plate, which places the surface later tested in sliding parallel to the deposited layers.

**Figure 1:**
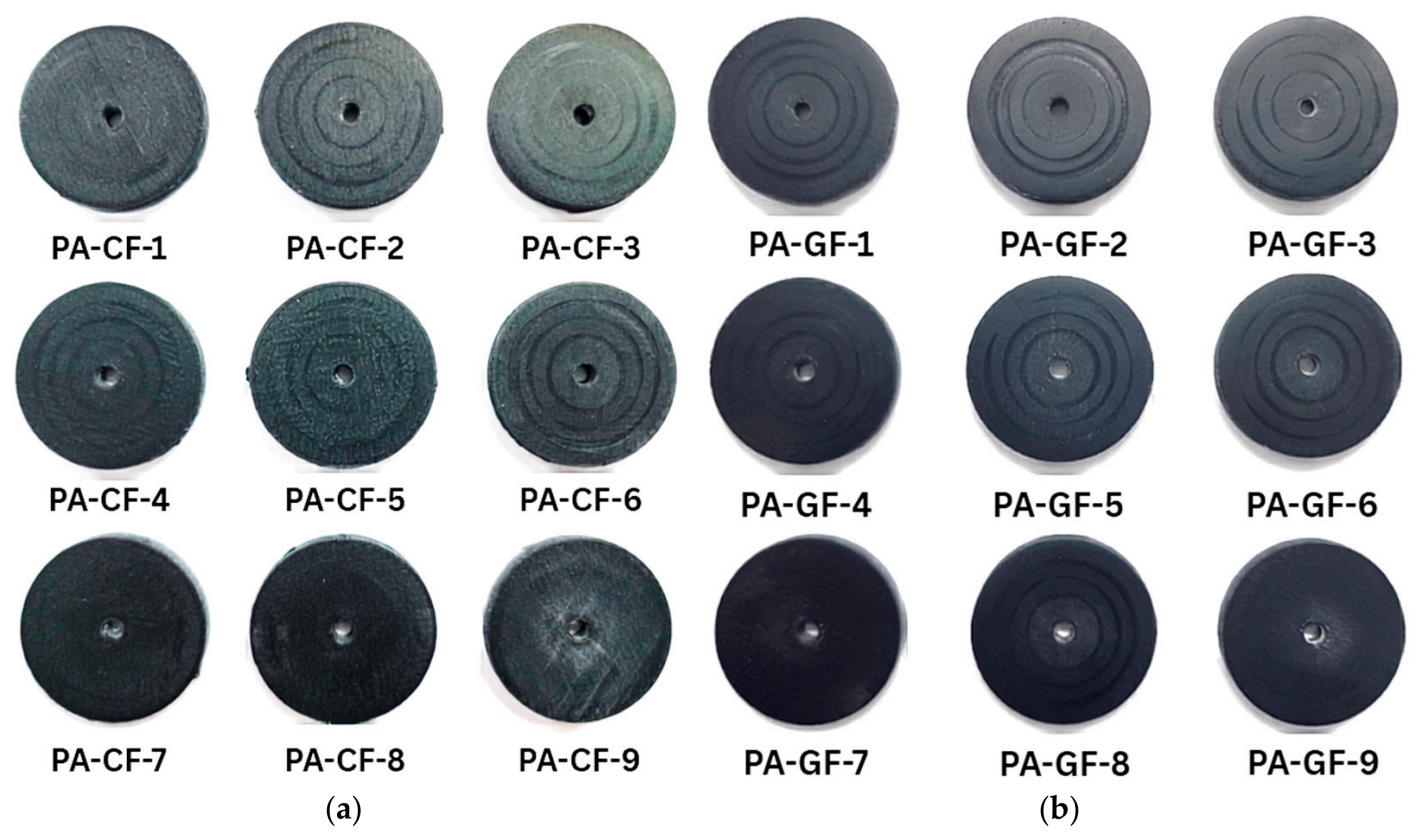
The disc specimens after tribological testing: (**a**) PA12-CF and (**b**) PA12-GF.

Raster orientation followed the alternating pattern set by default in the slicer and was neither varied nor recorded, since the surface preparation applied afterwards largely removed the as-printed texture. That preparation consisted of wet sanding with a progressive grit sequence up to 2000 grit, after which the discs were cleaned with technical alcohol to remove residual particles and debris. A final drying step, identical to the one applied before printing, removed the moisture taken up during wet sanding. The complete set of 18 specimens is shown in Figure 1.

### 2.3. Surface Roughness Measurements (Pre-Test)

The polished discs were characterized before tribological testing, following the sanding and polishing procedure described in Section 2.2, to establish the condition of the surface on which each track was subsequently run. Measurements were performed using a Taylor Hobson Surtronic S-100 stylus profilometer (Taylor Hobson Ltd., Leicester, UK) [20]. The arithmetic mean roughness (Ra) was determined in accordance with ISO 21920-2:2021 [21], using a cutoff length of 4 mm. Six readings were taken on each disc, distributed around the circumference at fixed angular spacing and oriented radially, so that any circumferential variation introduced during polishing would be captured rather than masked by a single localized measurement. The mean Ra of each disc was calculated from these readings.

Two considerations motivated this characterization. It confirmed that polishing produced a consistent finish across the discs of both materials, independent of the as-printed texture beneath. It also provided a per-disc record against which any variability later observed in friction and wear could be checked, since an unusually rough or smooth starting surface would offer a plausible explanation for a disc differing from its replicates.

### 2.4. Tribological Testing

Tribological tests were performed on an Anton Paar TRB3 tribometer (Anton Paar GmbH, Graz, Austria) [22] in a ball-on-disc configuration, in general accordance with ASTM G99 [23]. Friction and normal force were acquired at 80 Hz through the tribometer’s InstrumX data acquisition software, version V10.0.x [24]. The counterface was a 12.7 mm diameter ball of 100Cr6 bearing steel with a hardness of approximately 60 HRC, and a normal load of 20 N was applied and held fixed throughout.

Placing tracks at radii of 6.4, 9.6 and 12.7 mm on the same disc gave nominal sliding speeds of 0.5, 0.75 and 1.0 m/s, obtained by setting the rotational speed appropriate to each radius. The tracks are denoted V1, V2 and V3 in order of increasing radius. Because speed was varied by changing track radius rather than rotational speed at a fixed radius, sliding speed and track radius were confounded by design and cannot be statistically separated. Each track was run for 60 min, and the sliding distance was computed from the set speed and duration rather than from the raw distance output of the tribometer software. Disc mass was recorded before and after each test on an analytical balance with a resolution of 0.1 mg (0.0001 g). Before each post-test weighing, the disc surface was wiped with isopropyl alcohol to remove residual surface lubricant, no separate drying step was applied, and discs were weighed shortly after the end of each test.

Each material was tested under dry sliding and under each of the two protic ionic liquid lubricants introduced in Section 1.3, denoted MSu and MCi. For the lubricated tests, 20 µL of lubricant was distributed along the track radius with a micropipette immediately before starting, and a further 10 µL was added at 10, 30 and 50 min to compensate for depletion over the 60 min duration. Testing took place in a clean room under controlled and continuously monitored temperature and humidity, in accordance with ISO 291 and ISO/IEC 17025 [25,26]. The reported coefficient of friction for each test is the mean over a steady-state window that excludes both the initial run-in transient and the brief transients following each of the three lubricant top-ups (10, 30 and 50 min).

Tests were conducted in a fixed sequence, all PA12-CF discs (Dry, then MSu, then MCi conditions, in that order) were tested before all PA12-GF discs, with discs tested in ascending numerical order within each condition and track order being alternated between discs as described above. A new counterface ball was used for every individual test, and both the ball and disc were cleaned between tests.

### 2.5. Frictional Contact Temperature Monitoring

Temperature at the sliding contact was monitored throughout each test with a FLIR E30 infrared thermal imaging camera (Teledyne FLIR, Wilsonville, OR, USA) [27]. The ambient value near the tribometer was recorded in parallel, so that any rise at the contact could be interpreted against the surrounding environment rather than in isolation. An initial reading was taken immediately before the tribometer was started, and further readings followed at 10, 20, 30, 40, 50 and 60 min, covering the full duration of each track.

Polyamide 12 is a hygroscopic, semi-crystalline thermoplastic whose near-surface mechanical behavior can be sensitive to localized heating under extended sliding. Monitoring the contact therefore served two ends. It established whether frictional heating reached levels capable of influencing the material during a test, and it allowed differences in thermal evolution between materials, speeds and lubrication conditions to be set alongside the corresponding friction and wear results.

### 2.6. Wear-Rate Determination

Wear rate was determined from the wear track area (WTA), measured at six circumferential positions along each track with the Taylor Hobson Surtronic S-100 stylus profilometer described in Section 2.3 [20]. The mean of these measurements was converted to removed volume V, as follows:

(1) $$V={\mathrm{WTA}}_{\mathrm{mean}}\cdot 2\cdot \pi \cdot R.$$

where R is the track radius (mm). The specific wear rate, ws, was then obtained as follows:

(2) $$\mathrm{ws}=\frac{V}{F\cdot d}$$

where F is the applied normal load (N) and d is the sliding distance (m), calculated from the set sliding speed and test duration.

Mass change was recorded for every test but is not used as the primary wear-rate metric; specific wear rate is determined profilometrically throughout. Under dry sliding, the wear-predicted mass loss, calculated from the profilometric wear volume, is typically comparable to or below the 0.1 mg balance resolution, so the sign and magnitude of individual dry-test Δm values should not be interpreted as a reliable measure of wear. Under lubricated conditions, by contrast, the recorded mass changes substantially exceed this resolution and are discussed in Section 4.2 as evidence of lubricant/moisture uptake.

### 2.7. SEM/EDX Characterization

Worn surfaces were examined by scanning electron microscopy (SEM) using a JEOL JSM-5600LV instrument (JEOL Ltd., Tokyo, Japan) equipped with an Oxford Instruments energy dispersive X-ray (EDX) system (Oxford Instruments, Abingdon, UK) [28,29]. One disc per material and lubrication condition was selected, based on wear track visibility and surface clarity. The intention was to provide a representative view of the dominant damage mechanisms under each condition rather than an exhaustive survey of every track.

Examination was confined to the outermost wear track of each selected disc, at a radius of 12.7 mm and a nominal sliding speed of 1.0 m/s. Micrographs were acquired at nominal magnifications of 50, 100, 500 and 1000 times, capturing both the overall track morphology and the finer surface features. EDX spectra were recorded over the imaged fields, together with elemental maps of the same fields, to characterize the corresponding surface composition.

The steel counterbody was examined after selected tests under a digital optical microscope, in the as-tested condition and without cleaning, to preserve any material adherence within the contact zone. This examination was qualitative, and no dimensional measurements derived from it are reported.

### 2.8. Statistical Analysis

The effects of materials, lubricant condition and sliding speed on the tribological response were investigated using a full factorial experimental design. The experimental parameters were two composite materials, three lubricant conditions and three sliding speeds. Three independent disks were tested for each Material × Lubricant combination, thus totaling 18 disks (specimens). Each disk was tested for three tribological tests on three different tracks at different radial locations. The three sliding velocities were obtained from different radial positions but the same rotational velocity. Thus, each disk provided three replicate observations, for a total of 54 disc-track observations for each response variable.

The three sliding-speed measurements were not treated as statistically independent, as they came from different tracks of the same disk. Thus, the disk was considered as a random experimental unit, and the three track measurements were repeated measurements on the same disk. This structure was included in a linear mixed-effects model to account for the correlation between measurements within the same disk.

Statistical analysis was carried out using Minitab 19 software (Coventry, UK). The material, lubricant condition and sliding speed were fixed factors. All two-way interactions and the three-way interaction were entered into the model. The disk effect was specified as a random effect nested within the Material × Lubricant combination.

The significance of the fixed effects was tested with F-tests at a significance level of alpha = 0.05. Where significant interactions were found, the corresponding interaction effects were further explored using interaction plots and suitable multiple-comparison methods. The PC values were used to describe the relative contribution of the model terms with qualitative descriptors (e.g., highly significant, significant and insignificant), assigned on the basis of the corresponding F-statistic and *p*-value. The adequacy of the model was verified using residual analysis, including checking the normality and constant variance assumptions.

## 3. Results

### 3.1. Coefficient of Friction

**Figure 2:**
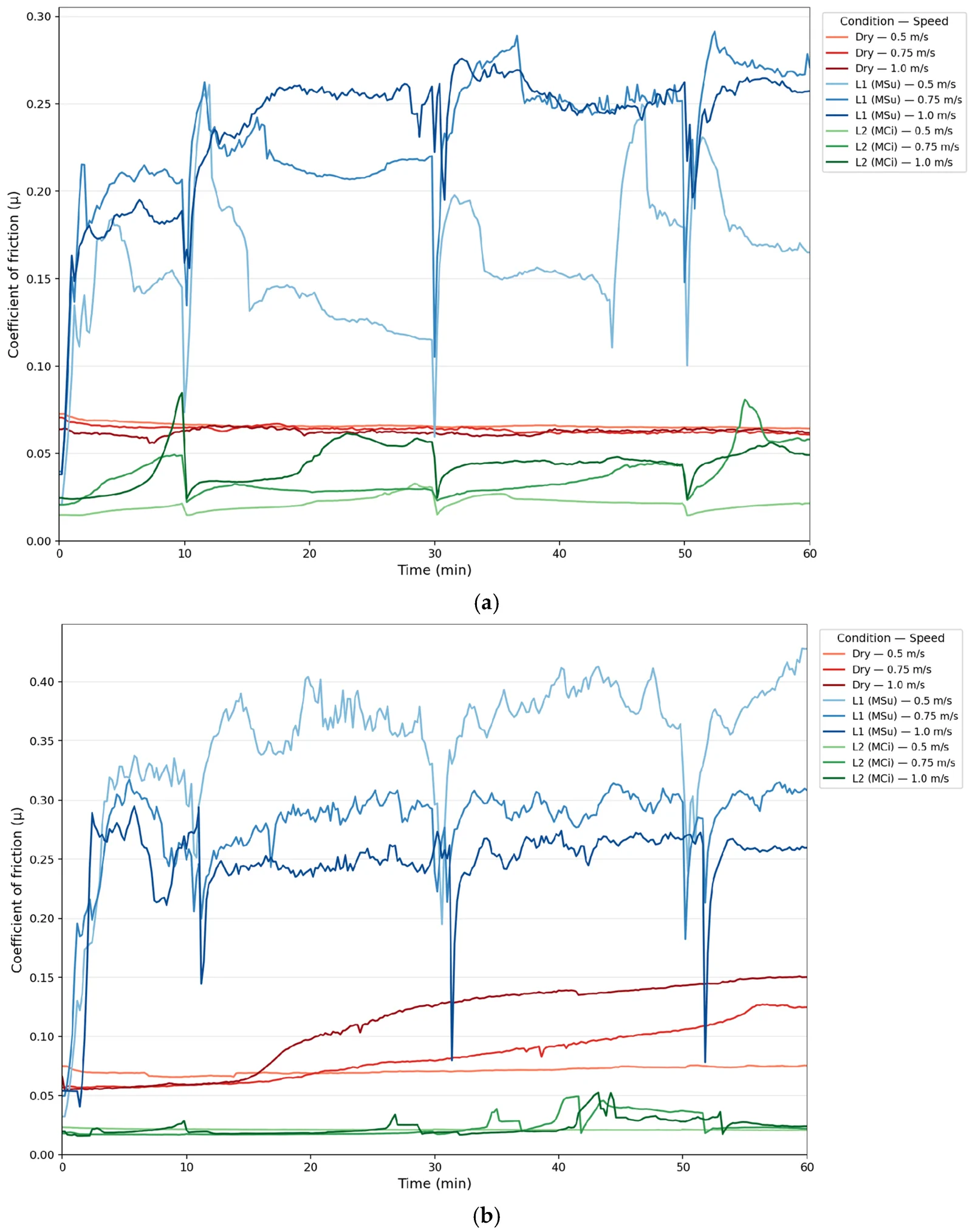
Coefficient of friction as a function of time, averaged across replicate discs, for each lubrication condition (Dry, L1/MSu, L2/MCi) and sliding speed (0.5, 0.75, 1.0 m/s), for (**a**) PA12-CF and (**b**) PA12-GF.

The coefficient of friction, specific wear rate, surface roughness and temperature are reported in Table 2, and the corresponding time-resolved traces, averaged across discs for each material, condition and sliding speed, are presented in Figure 2. Under dry sliding, the coefficient of friction fell between 0.05 and 0.08 for PA12-CF and between 0.06 and 0.15 for PA12-GF, with the traces staying largely flat over the 60 min duration at every speed tested.

At the level of individual tests, the coefficient of friction under dry sliding rose with speed on discs GF-D-01 and GF-D-11, reaching 0.1500 on GF-D-11-V3, the highest value recorded under this condition. GF-L1-13-V1 exhibited the highest value of the whole study (0.3522). The L1 condition always gave higher COF values than dry sliding for both materials. A significant increase in the viscosity of the lubricant was observed. Under MCi, test CF-L2-07-V3 reached 0.0755 against a group mean of 0.0439, the largest departure observed under this lubricant.

Lubrication with MSu (L1) raised the coefficient of friction substantially above the dry baseline for both materials, to between 0.12 and 0.29 for PA12-CF and between 0.22 and 0.35 for PA12-GF. In most L1 tests the trace climbed progressively over 60 min rather than holding steady, an effect most pronounced at 0.5 m/s for both materials (Figure 2).

MCi (L2) produced the opposite effect. Friction fell well below the dry baseline for both materials, with a mean of 0.023 for both materials, an order of magnitude below the corresponding L1 values.

Comparing the two materials, MCi brought them to the same level, with a mean of 0.0225 for PA12-CF and 0.0226 for PA12-GF. Under dry sliding, they overlapped at 0.5 m/s but separated at the higher speeds, with PA12-GF reaching 0.071 to 0.150 at 1.0 m/s against 0.051 to 0.075 for PA12-CF. Under MSu, the glass fiber composite gave consistently higher friction throughout. The friction-increasing effect of that lubricant was therefore more pronounced for PA12-GF, and the material also separated the two composites under dry sliding once the speed exceeded 0.5 m/s.

### 3.2. Pre-Test Surface Roughness

Following the sanding and polishing procedure described in Section 2.2, the mean pre-test roughness (Ra) of the nine PA12-CF discs was 0.264 ± 0.019 µm, spanning 0.242 to 0.298 µm across individual specimens. The nine PA12-GF discs gave 0.263 ± 0.012 µm, spanning 0.247 to 0.277 µm. Per-disc values are reported alongside the friction and wear data in Table 2. The two materials therefore agree closely, both in mean and in the narrow spread across replicates. Preparation produced a consistent starting condition regardless of reinforcement type, so differences in friction and wear observed between the composites cannot be attributed to the state of the surface before testing.

### 3.3. Wear Rate

Specific wear rates for all individual tests are reported in Table 2. For PA12-CF, the mean was 1.83 × 10^−6^ mm^3^/(N·m) under dry sliding, rising to 14.77 × 10^−6^ mm^3^/(N·m) under MSu and falling to 1.34 × 10^−6^ mm^3^/(N·m) under MCi. The corresponding figures for PA12-GF were 2.81, 6.43 and 2.34 × 10^−6^ mm^3^/(N·m).

Both materials therefore ranked the lubrication conditions identically, with MSu giving the highest wear rate, MCi the lowest, and dry sliding an intermediate value. The separation was far wider for PA12-CF, where the mean under MSu exceeded that under MCi by a factor of eleven, against less than three for PA12-GF. The ranking of the materials, by contrast, depended on the condition. PA12-GF wore faster under dry sliding and under MCi, while PA12-CF wore faster under MSu and by a wider margin. Material type therefore did not determine the higher-wearing material independently of the lubricant.

### 3.4. Frictional Contact Temperature

**Figure 3:**
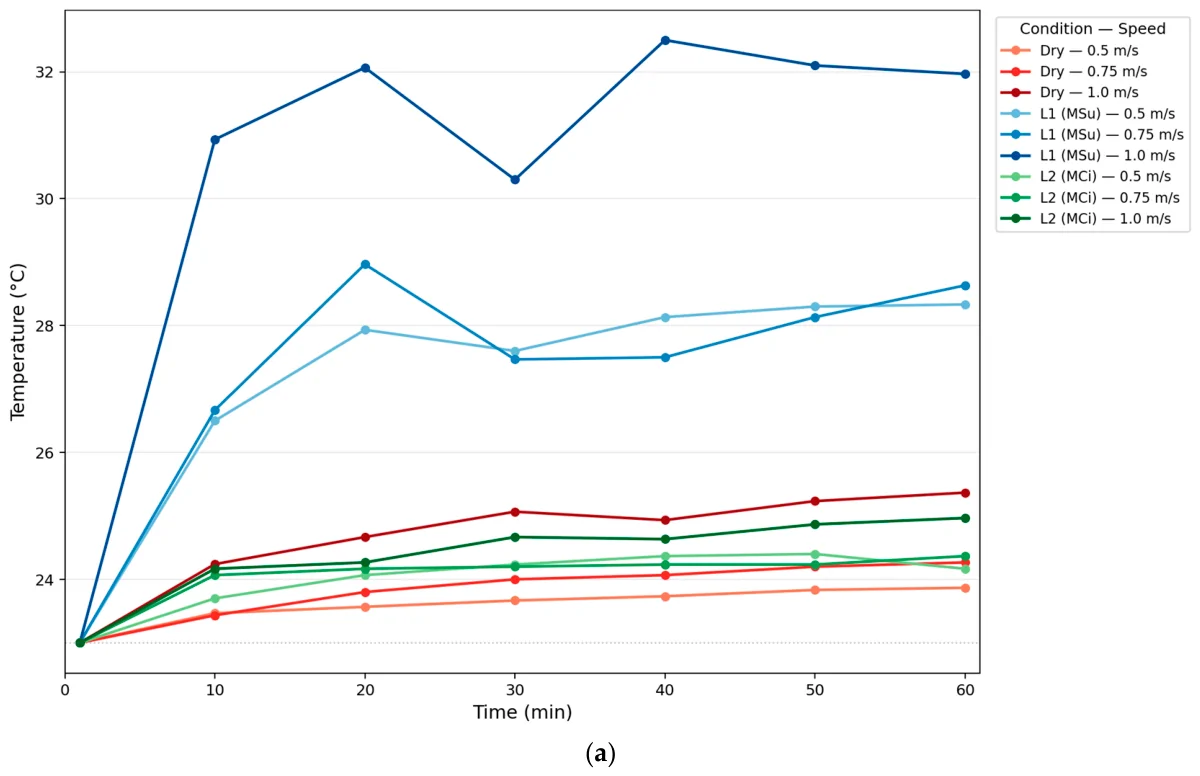

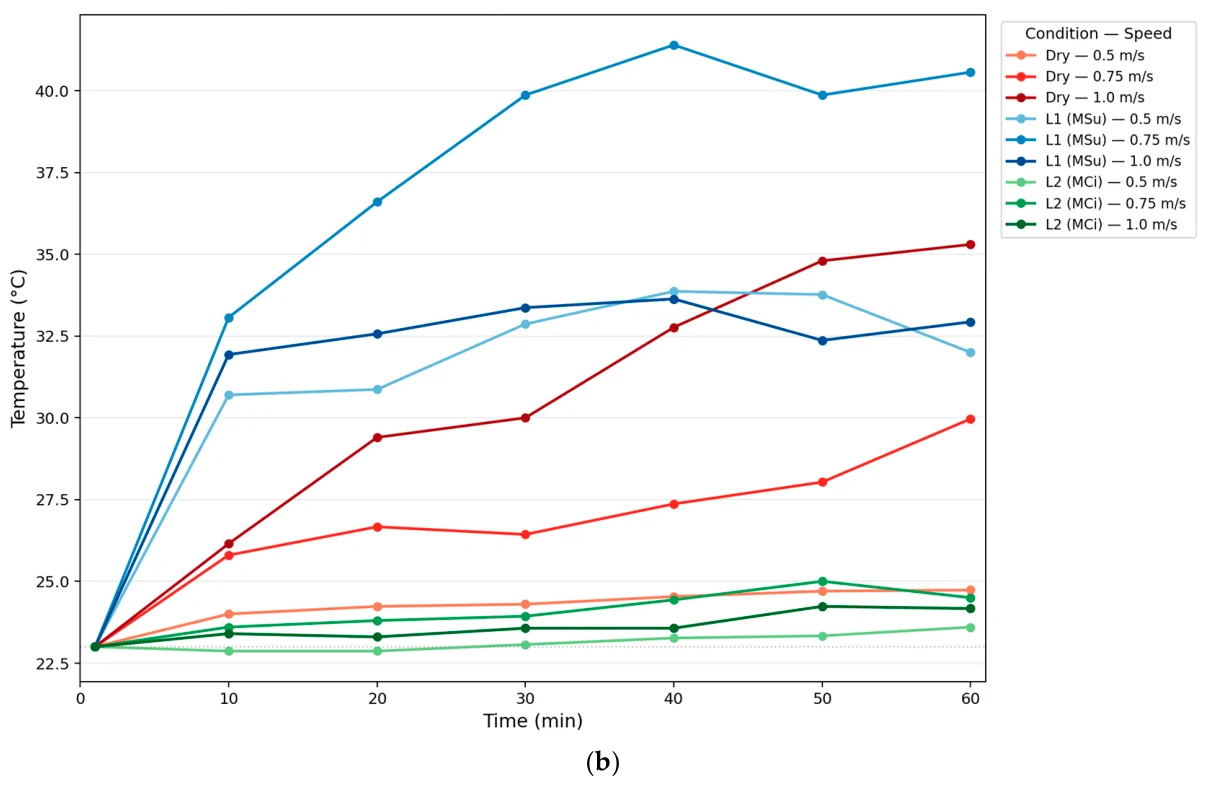
Contact-point temperature as a function of time, averaged across replicate discs, for each lubrication condition (Dry, L1/MSu, L2/MCi) and sliding speed (0.5, 0.75, 1.0 m/s), for (**a**) PA12-CF and (**b**) PA12-GF.

Contact-point temperature over the 60 min duration, averaged across replicate discs for each material, condition and sliding speed, is presented in Figure 3. Under MCi, both materials stayed within roughly two degrees of the 23 °C starting value at every speed. PA12-CF behaved similarly under dry sliding, where the mean rise did not exceed 2.4 °C even at 1.0 m/s.

MSu produced a markedly different thermal response in both materials. Temperature climbed well above the dry and MCi baselines within the first 10 to 20 min and then largely stabilized for the remainder of the test. For PA12-CF, the mean rise reached 9.0 °C at 1.0 m/s and between 5.3 and 5.6 °C at the two lower speeds. PA12-GF ran hotter still, averaging 9.0 °C at 0.5 m/s and peaking at 17.6 °C at 0.75 m/s before falling back to 9.9 °C at the highest speed.

PA12-GF also departed from PA12-CF under dry sliding, where the mean rise grew with speed from 1.7 °C at 0.5 m/s to 7.0 °C at 0.75 m/s and 12.3 °C at 1.0 m/s. The corresponding values for PA12-CF were 0.9, 1.3 and 2.4 °C.

The two materials therefore separate thermally under dry sliding as soon as the speed exceeds 0.5 m/s, while remaining comparable under MCi throughout. Dispersion between replicate discs was also considerably larger for PA12-GF under dry sliding at 1.0 m/s, where individual values ranged from 3.2 to 20.2 °C.

### 3.5. SEM/EDX Analysis of the Worn Surfaces

**Figure 4:**
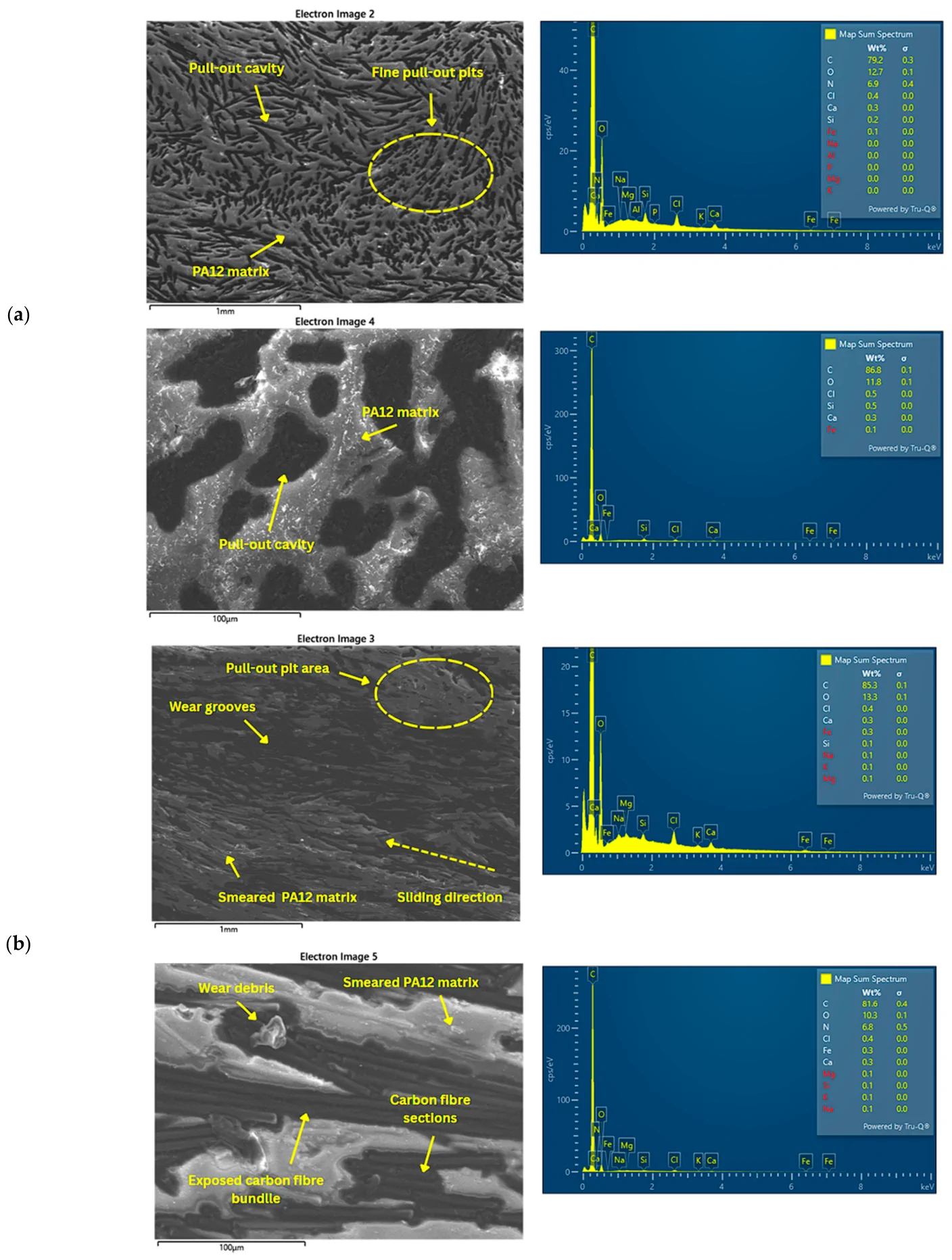

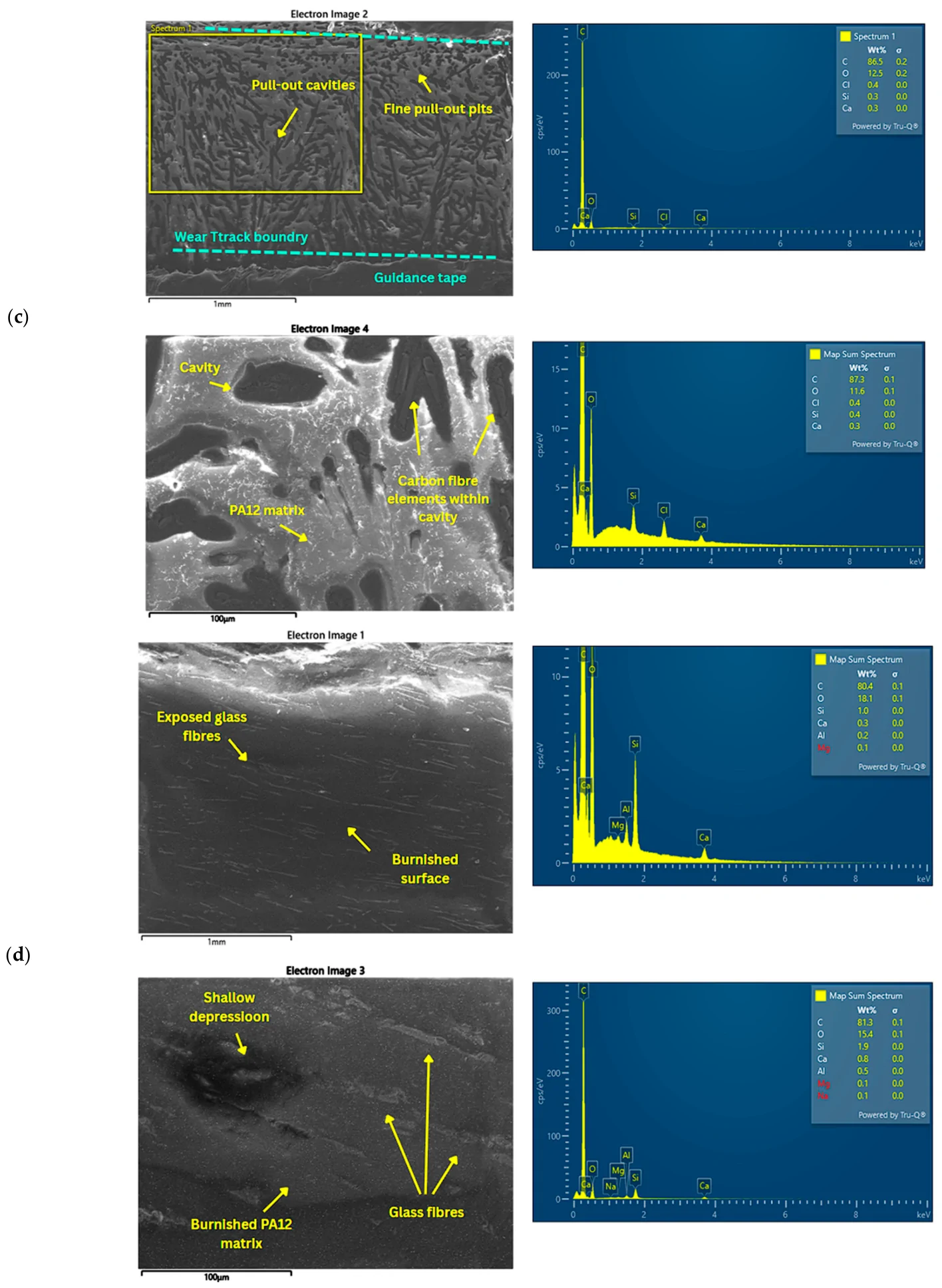

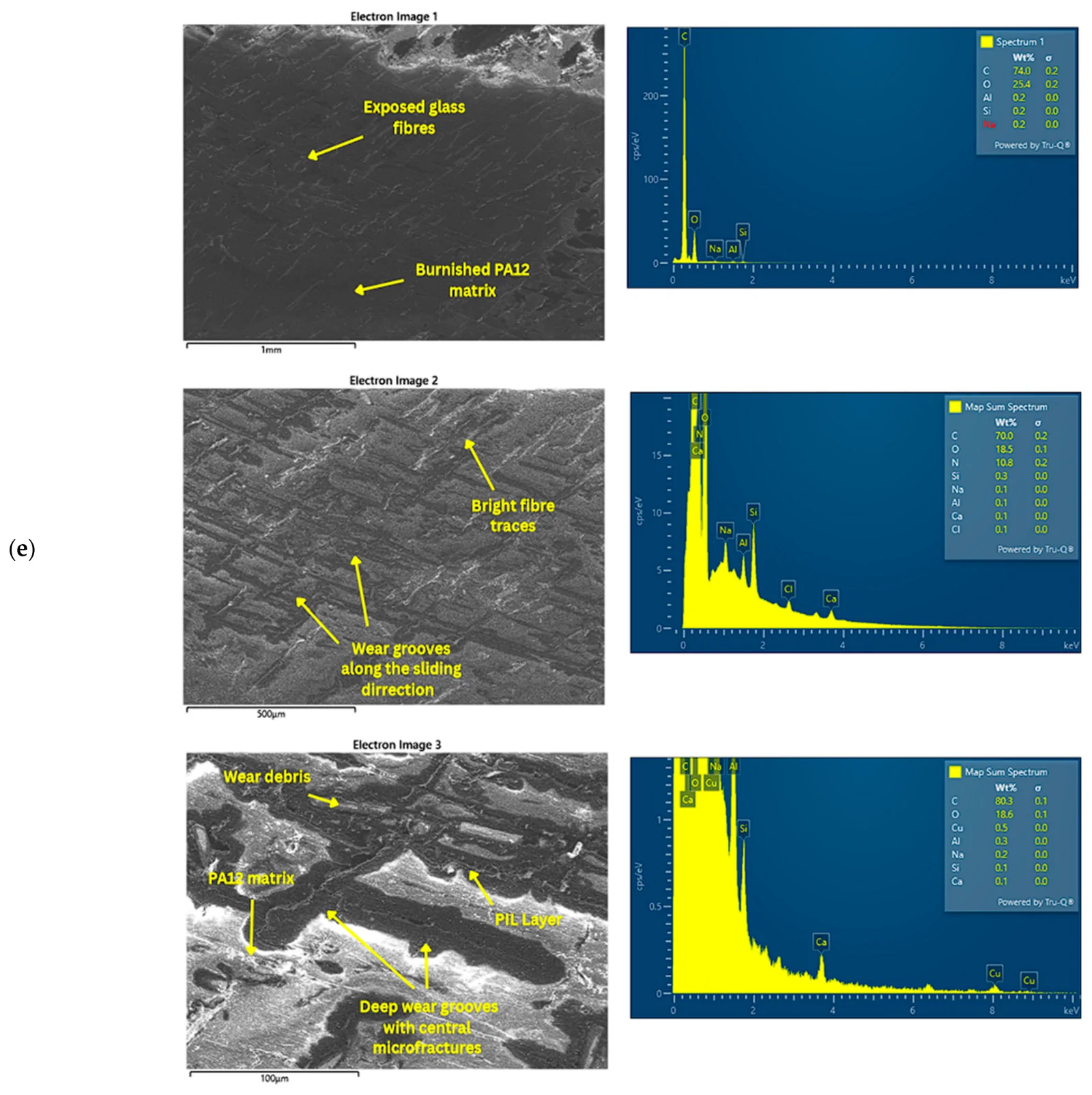

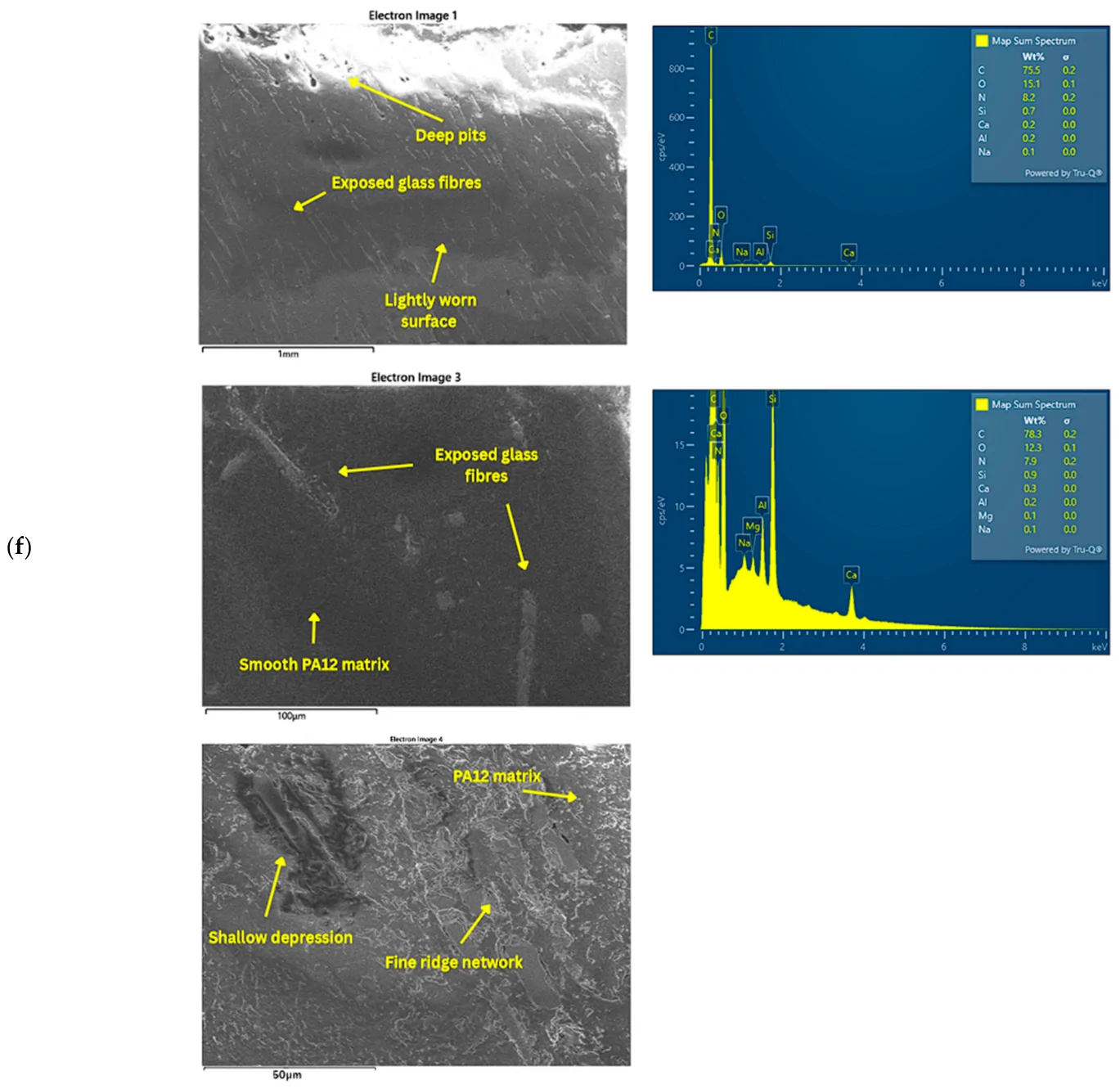
SEM micrographs and corresponding EDX spectra of representative worn surfaces, for PA12-CF under (**a**) dry sliding, (**b**) L1 (MSu) lubrication and (**c**) L2 (MCi) lubrication, and for PA12-GF under (**d**) dry sliding, (**e**) L1 (MSu) lubrication and (**f**) L2 (MCi) lubrication.

Representative worn surfaces of both composites, examined under each lubrication condition, are presented in Figure 4. The PA12-CF discs appear in Figure 4a for dry sliding, Figure 4b for L1 and Figure 4c for L2, and the PA12-GF discs appear in Figure 4d–f in the same order. Each panel combines micrographs acquired at increasing magnification with the associated EDX spectrum, so that wear track morphology and finer surface features can be assessed alongside the corresponding elemental composition.

Under dry sliding, the PA12-CF wear track in Figure 4a is covered by a dense population of dark, elongated cavities extending across the entire field, with their long axes distributed over a range of directions. At higher magnification, the cavities resolve as recessed openings separating the intervening matrix, with their outlines irregular and broadened. Carbon and oxygen dominate the associated spectra, at 79.2 wt% C and 12.7 wt% O in the wider field and 86.8 wt% C and 11.8 wt% O in the closer one.

The same material under L1 lubrication, presented in Figure 4b, presents a markedly different topography. At the lower magnification, the surface is traversed by elongated wear grooves aligned along a common direction, corresponding to the sliding direction within the track. Individual carbon fibers become resolvable at higher magnification as parallel cylindrical bodies carrying longitudinal striations and grouped into bundles, while the intervening matrix forms smooth, lobed regions that extend over and partly cover them. Discrete wear debris particles are present on the track. The spectra give 85.3 wt% C and 13.3 wt% O in the wider field and give 81.6 wt% C, 10.3 wt% O and 6.8 wt% N in the closer one. Iron is quantified at 0.3 wt% in the latter, the only instance in the present study for which iron was reported above the significance threshold of the analysis software, indicating transfer of material from the 100Cr6 counterface onto the composite surface.

L2 lubrication returns the PA12-CF track, as presented in Figure 4c, to a population of dark, elongated cavities comparable in extent and in individual size to that seen under dry sliding. Their long axes, however, follow a different distribution of directions across the field. At higher magnification, the cavities appear narrower and more sharply outlined than their counterparts on the dry track. Several of the larger ones contain elongated bodies lighter than the surrounding cavity floor, consistent with reinforcement retained within the cavity rather than removed from it. The spectra give 86.5 wt% C and 12.5 wt% O in the wider field and 87.3 wt% C and 11.6 wt% O in the closer one, with no counterface element detected.

The PA12-GF wear tracks present a consistently different topography. Under dry sliding, as presented in Figure 4d, the track is smooth and burnished over most of the field, without the cavity network that characterizes PA12-CF under the same condition, and carries fine elongated features brighter than the surrounding matrix. The burnished character is maintained at higher magnification, with only shallow depressions and isolated fine grooves interrupting the surface. Elemental mapping identifies the bright features as the glass reinforcement, since silicon, aluminum, calcium and oxygen concentrate within them, while the carbon signal does not. The spectra give 80.4 wt% C, 18.1 wt% O and 1.0 wt% Si in the wider field and give 81.3 wt% C, 15.4 wt% O, 1.9 wt% Si, 0.8 wt% Ca and 0.5 wt% Al in the closer one. Both the oxygen and the silicon contents exceed those measured on any PA12-CF disc, consistent with the oxide composition of the glass reinforcement. GF-D-11, the disc examined here for the dry condition, is separately identified in Section 4.5 as showing anomalously high friction and contact-point temperature at this same track. Disc selection for SEM/EDX was based on wear track visibility and surface clarity. This is a criterion independent of tribological performance, so the coincidence with the disc’s friction/thermal anomaly does not reflect a biased choice. The disc’s specific wear rate, however, measured profilometrically across all three replicate discs, falls within 8% of the other two dry PA12-GF discs at every speed tested, despite its elevated friction and temperature. Since wear rate is the profilometric proxy for the extent of material removal underlying the observed morphology, this provides independent evidence that the smooth, burnished topography reported here is unlikely to be an artifact specific to this disc’s anomalous behavior.

Under L1 lubrication, the PA12-GF track presented in Figure 4e appears comparatively smooth at the lower magnification, where shallow grooves traverse the surface without pronounced relief. The extent of the damage becomes apparent only at higher magnification, where cavities, PA12 matrix material and layered fragments are observed across the field. Of the PA12-GF specimens examined, this track carries the most developed surface damage. The associated spectra also differ from every other spectrum in the set. Carbon falls to 62.0 wt% in the wider field, while oxygen rises to 22.4 wt% and nitrogen to 15.2 wt%. The enrichment persists at intermediate magnification, with 70.0 wt% C, 18.5 wt% O and 10.8 wt% N, and at the highest, with 80.3 wt% C and 18.6 wt% O. Since MSu contains both oxygen and nitrogen in proportions considerably higher than those of polyamide 12, their simultaneous enrichment indicates lubricant retained on the worn surface.

The PA12-GF track under L2 lubrication, as presented in Figure 4f, is smooth and only lightly marked. At the lower magnification, the glass reinforcement is visible as bright elongated features oriented both along and transverse to the sliding direction, together with isolated deep pits. The surface remains largely featureless at intermediate magnification apart from individual fibers, and at the highest it carries a fine network of narrow ridges together with a shallow depression, without cavities or delaminated material. The spectra give 75.5 wt% C, 15.1 wt% O and 8.2 wt% N in the wider field and 78.3 wt% C, 12.3 wt% O and 7.9 wt% N in the closer one. The nitrogen content corresponds to that expected for the polyamide matrix, and no oxygen enrichment comparable to that under L1 is present, indicating that no appreciable lubricant residue remained.

Comparing the two composites, reinforcement type governs the topography of the worn surface under every lubrication condition examined. PA12-CF develops an open network of cavities throughout, while PA12-GF retains a comparatively smooth and burnished track in which the reinforcement remains embedded and level with the matrix. The contrast is narrowest under L1, where both materials show marked surface damage, and widest under L2, where the PA12-GF track is only lightly marked while PA12-CF still presents a developed cavity network. The chemical evidence follows the same division. Transfer of counterface material was detected only on PA12-CF, and lubricant was retained on the surface only on PA12-GF, both under L1 lubrication.

### 3.6. Optical Microscopy Analysis of the Counterbody

**Figure 5:**
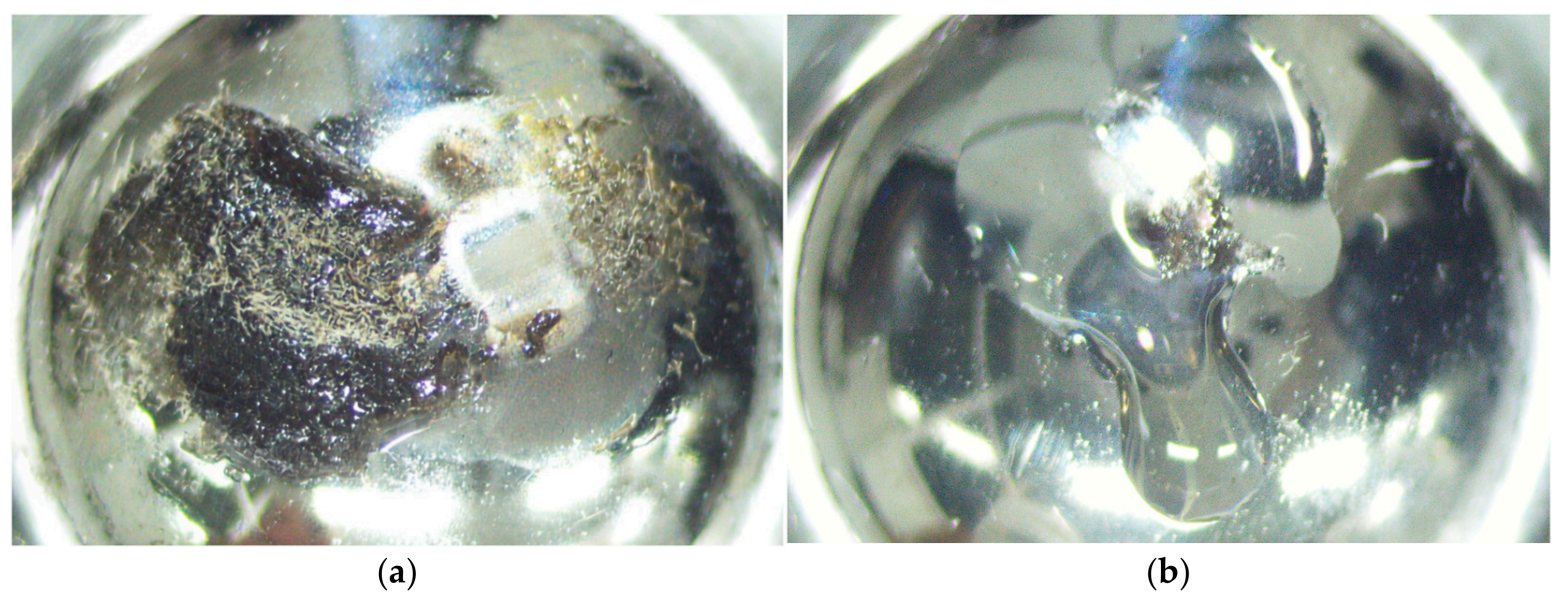
Optical micrographs of the 100Cr6 counterbody after testing, examined without cleaning, for (**a**) test CF-L1-06-V3, lubricated with MSu, and (**b**) test CF-L2-09-V3, lubricated with MCi.

The condition of the steel counterbody after testing is compared in Figure 5 for the two protic ionic liquid lubricants. Both images come from tests performed on the outermost track at 1.0 m/s against PA12-CF, so that the comparison isolates the effect of the lubricant. Figure 5a corresponds to test CF-L1-06-V3, lubricated with MSu, and Figure 5b to test CF-L2-09-V3, lubricated with MCi. The counterbodies were examined as tested, without cleaning, and the observations reported here are qualitative.

After sliding under MSu, the contact zone carries a dark, opaque deposit covering a substantial part of the loaded area. The deposit has a matte, granular texture and a clearly defined outline, standing out against the reflective steel around it. Fine-entrained material is visible along its margin, indicating that wear debris generated during the test became incorporated into the deposit rather than being carried out of the contact.

After sliding under MCi, the counterbody presents a markedly different appearance. The contact zone remains largely specular, and the lubricant is retained as thin translucent patches with rounded, lobed margins, consistent with a film that stayed fluid throughout. A localized cluster of fine granular material is present, but nothing resembling the consolidated deposit formed under MSu.

The contrast between the two counterbodies provides direct observational support for a change in the physical state of MSu during testing. Whereas MCi remained fluid and left only a thin film, MSu formed a consolidated deposit that accumulated wear debris over the course of the test. This agrees with the friction behavior reported in Section 3.1, where MSu produced coefficients of friction well above the dry baseline and traces that rose progressively over the 60 min duration. MCi, by contrast, produced values an order of magnitude lower and stable throughout. The surface composition reported in Section 3.5 points the same way, since lubricant retained on a worn surface was detected only under MSu.

### 3.7. Statistical Analysis Results

**Figure 6:**
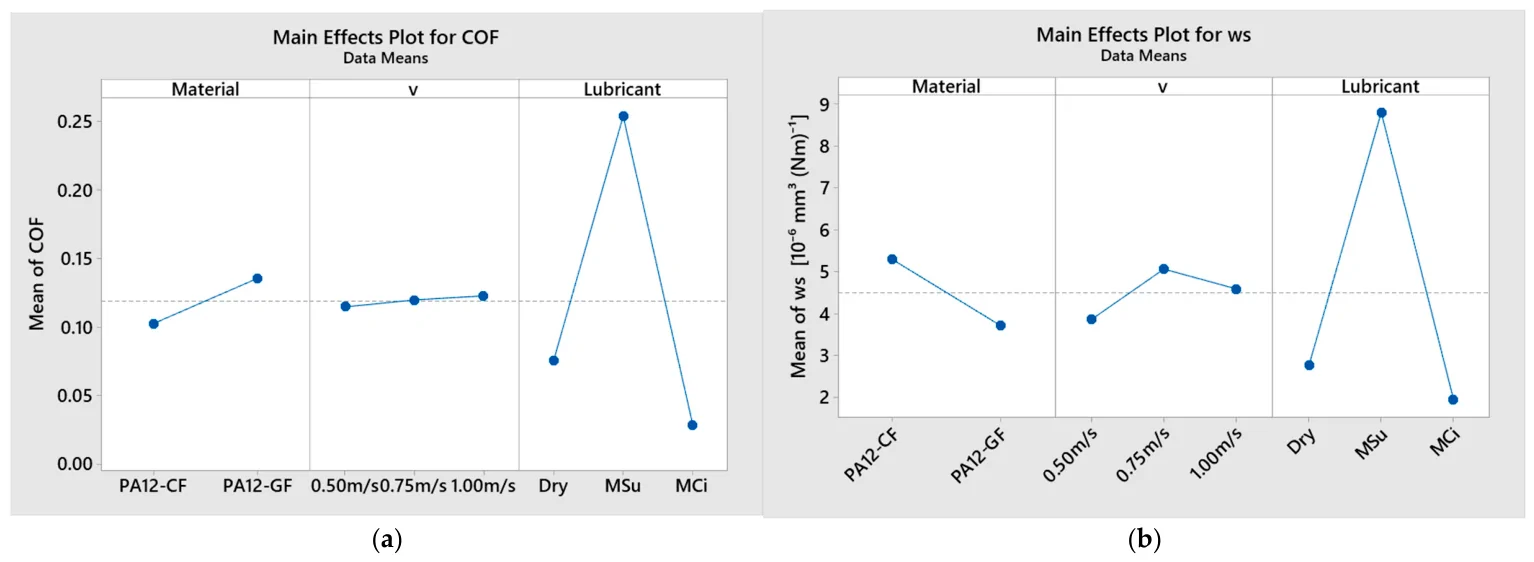
Main effects plots for (**a**) mean steady-state COF; (**b**) specific wear rate, ws.

**Figure 7:**
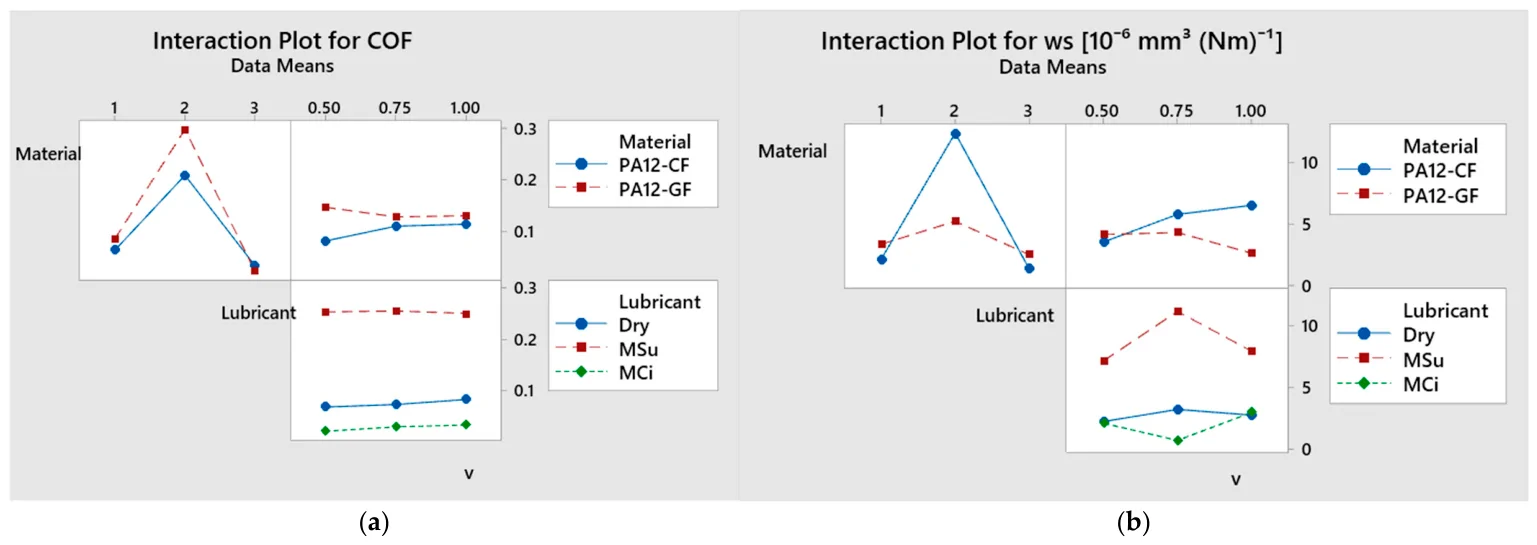
Interaction plot for (**a**) COF and (**b**) ws.

**Figure 8:**
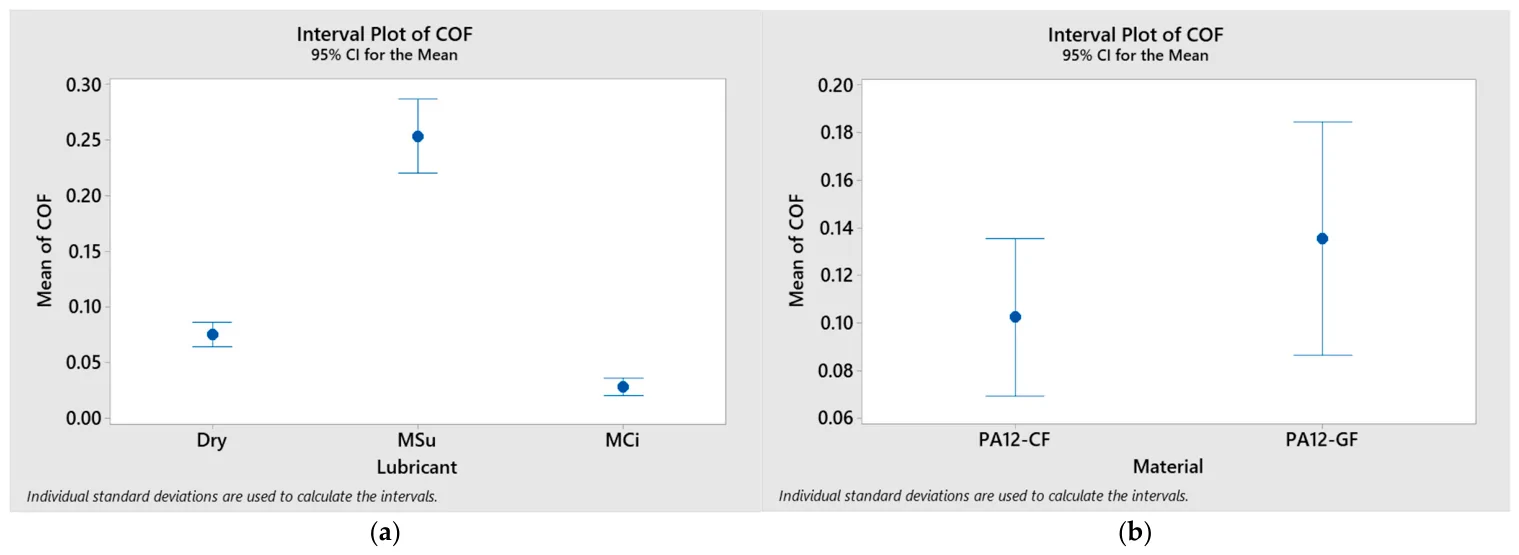
Interval plot for COF: (**a**) lubricant; (**b**) material.

**Figure 9:**
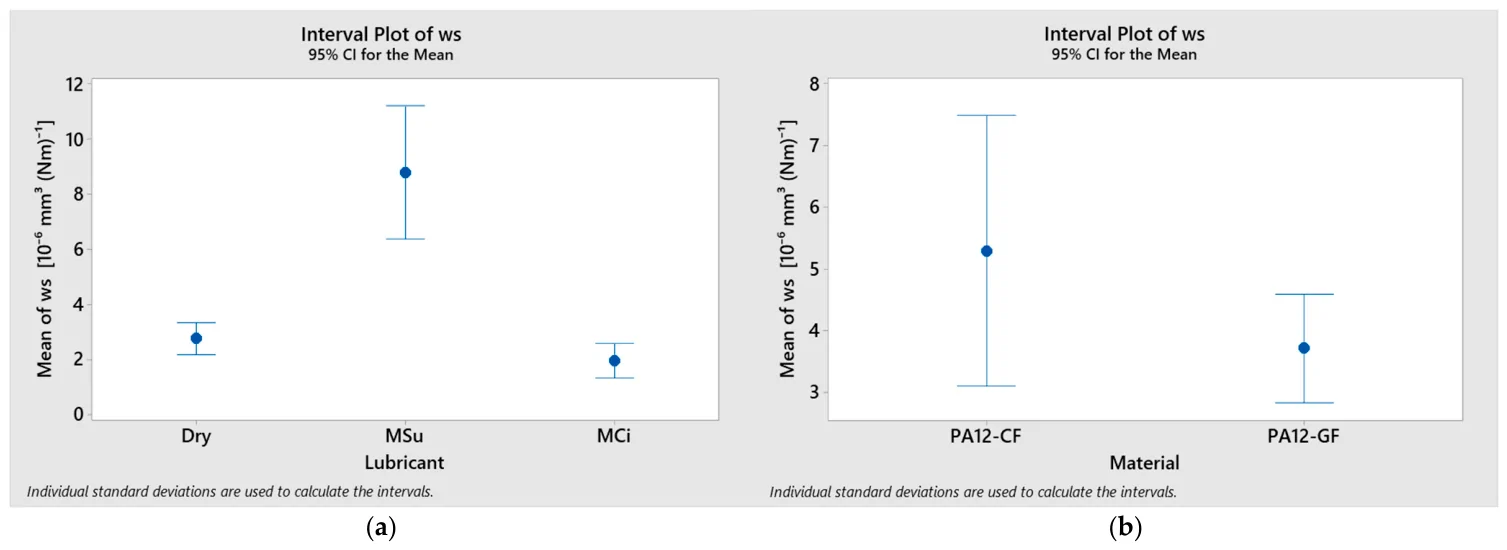
Interval plot for ws: (**a**) lubricant; (**b**) material.

**Figure 10:**
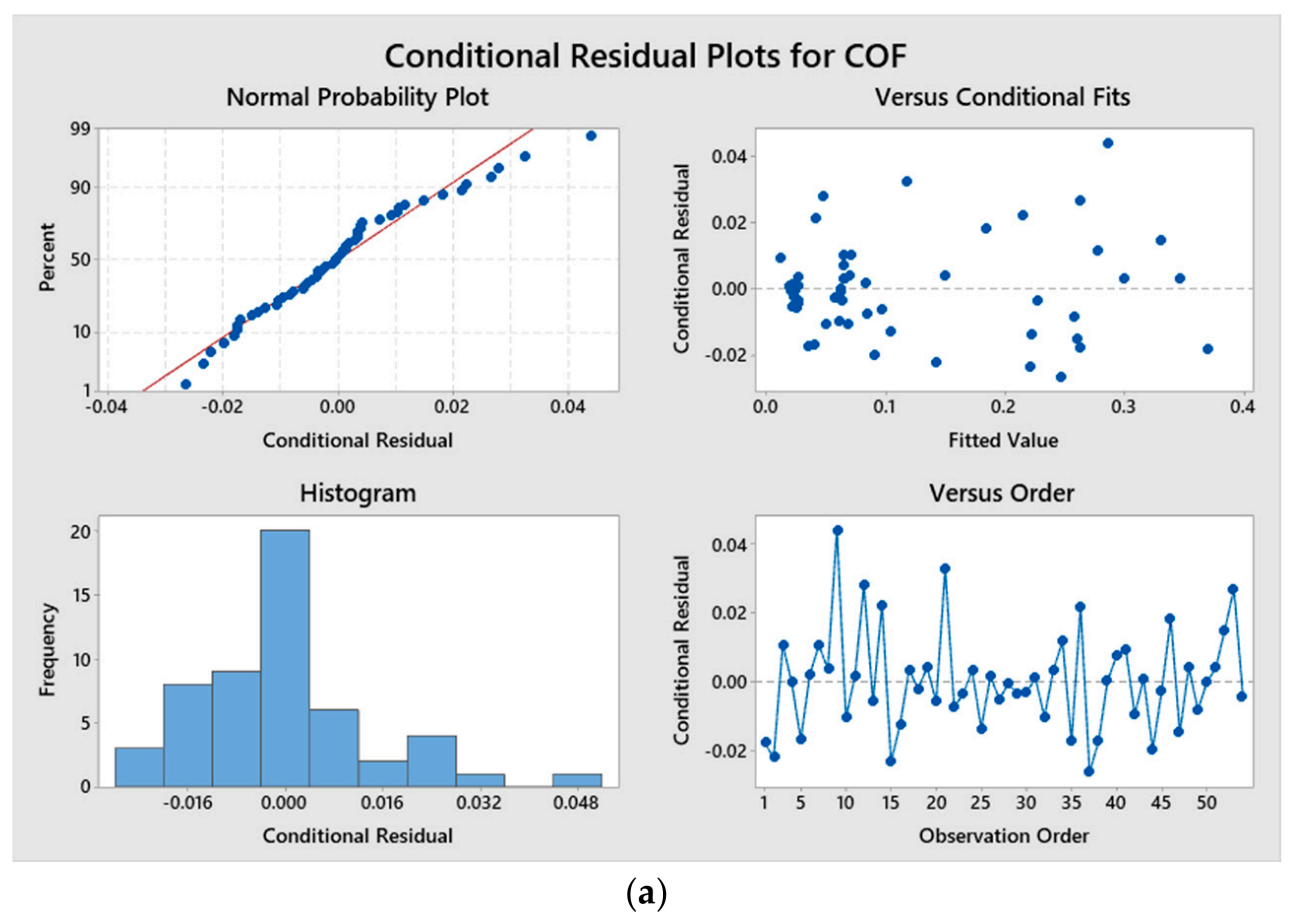

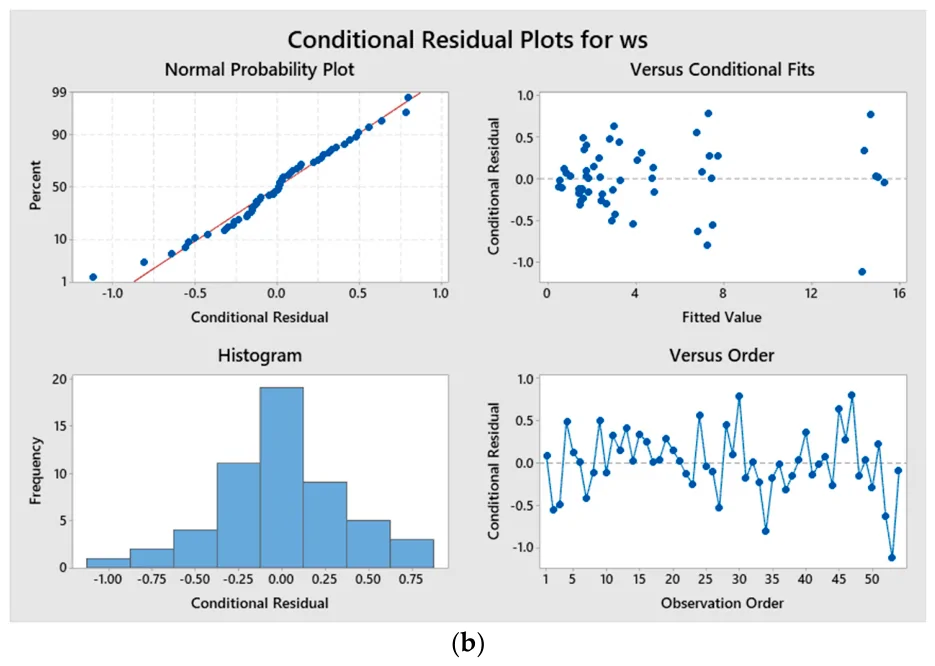
Residual plots for (**a**) COF and (**b**) ws.

The results of the statistical analysis, the mixed-effects analysis table and the statistical graphics for COF and ws are shown in Table 3 and Table 4 and Figure 6, Figure 7, Figure 8, Figure 9 and Figure 10, respectively.

The mixed-effects analysis showed significant effects of material and lubricant condition on COF (Table 3), whereas the overall effect of sliding speed was not statistically significant. The material effect was significant (F_1,12_ = 11.85, *p* = 0.005), while the lubricant effect was highly significant (F_2,12_ = 203.43, *p* < 0.001). By contrast, sliding speed did not have a significant main effect (F_2,24_ = 0.75, *p* = 0.484).

The overall effect of sliding speed was not significant, but there were significant interactions with speed. The interaction Material × Sliding Speed was significant (F_2,24_ = 8.65, *p* = 0.001), indicating that the change in COF with sliding speed was different for different materials. More significantly, the three-way interaction of Material × Lubricant × Sliding Speed was very significant (F_4,24_ = 11.24, *p* < 0.001). The influence of sliding speed on COF can thus not be understood in isolation of the material and lubricant condition.

The Material × Lubricant interaction was also significant (F_2,12_ = 9.31, *p* = 0.004), demonstrating that the influence of the lubricant condition on COF depended on the material. In contrast, the Sliding Speed × Lubricant interaction was not statistically significant (F_4,24_ = 0.39, *p* = 0.816) when averaged over the two materials.

The mixed-effects models also provided information on the variation between disks (random-effect contribution). The random disk component for COF was estimated at 0.000284, which was 41.44% of the total variance, while the residual component was 58.56%. The between-disc component was non-significant in the variance-component test (*p* = 0.052), but the magnitude of the estimated between-disc component shows considerable disc-to-disc variability. For specific wear rate, the estimated variance due to the disk was 0.052855, which accounted for 18.12% of the total variance, and the remaining 81.88% was due to residual variability. Block variance was not statistically significant (*p* = 0.184). However, the random effect at the disk level was retained because the experimental design involved three measurements from different tracks of the same disk.

The mixed-effects analysis of the specific wear rate (Table 4) showed a substantially stronger dependence on the experimental factors than that observed for COF. All three main effects were statistically significant: material (F_1,12_ = 85.13, *p* < 0.001), sliding speed (F_2,24_ = 27.80, *p* < 0.001), and lubricant condition (F_2,12_ = 635.60, *p* < 0.001).

Moreover, all interaction terms were highly significant. The interaction Material × Sliding Speed was significant (F_2,24_ = 94.68, *p* < 0.001), as was the interaction Material × Lubricant (F_2,12_ = 262.17, *p* < 0.001). Also, the interaction between Sliding Speed and Lubricant was highly significant (F_4,24_ = 62.95, *p* < 0.001). Crucially, the three-way interaction Material × Lubricant × Sliding Speed was highly significant (F_4,24_ = 88.15, *p* < 0.001). These results show that the specific wear rate is controlled by a strong combined effect of the material, lubricant condition and sliding speed.

Figure 7a shows the interaction plots for COF, which further elaborate these effects. The fitted means show that lubricant MSu gave significantly higher COF values than lubricant dry conditions and MCi for both materials. For lubricant MSu, the fitted COF increased from about 0.21 for PA12-CF to about 0.30 for PA12-GF. Conversely, COF values were the lowest for lubricant MCi, with fitted means of ~0.03 for PA12-CF and ~0.02 for PA12-GF. Dry condition gave intermediate values, of about 0.065 and 0.085 for PA12-CF and PA12-GF, respectively. Thus, the effect of material on COF was dependent on the lubricant condition. PA12-GF exhibited a higher COF compared to PA12-CF under Dry and MSu conditions, but the difference between materials was reversed under MCi. This change in the relative material response is in agreement with the significant Material × Lubricant interaction. The fitted means also indicate that the effect of sliding speed on COF was relatively small when averaged over all lubricant conditions, consistent with the non-significant main effect of speed.

The Material × Lubricant plot shows that lubricant MSu generated considerably higher specific wear rates than dry and MCi conditions (Figure 7b). For PA12-CF, the fitted wear rate under lubricant MSu was approximately 12.3, compared with approximately 2.1 and 1.4 for the dry condition and MCi, respectively. For PA12-GF, the corresponding fitted values were approximately 5.1, 3.3, and 2.5. Thus, lubricant MSu resulted in a particularly pronounced increase in wear rate for Material 1.

The effect of the lubricant was therefore highly material-dependent. While the difference of the materials was rather small under lubricant MCi, lubricant MSu led to a significantly higher wear rate for PA12-CF compared to PA12-GF. This behavior is reflected by the highly significant Material × Lubricant interaction.

The fitted means also show strong dependence of wear rate on sliding speed. When averaged over the other factors, the response was nonlinearly dependent on speed. But the Material × Speed interaction reveals that the direction and magnitude of this variation depended on the material. The fitted wear rate for PA12-CF increased from 3.6 to 6.6 at v = 0.50 and v = 1.00, respectively. However, PA12-GF responded differently, with a maximum wear rate of 4.4 at v = 0.75 and a minimum wear rate of 2.7 at v = 1.00.

The Speed × Lubricant interaction also supports the idea that the effect of speed depended on the lubricant. For the lubricant MSu, the fitted wear rate increased significantly, from about 7.3 at v = 0.50 to about 11.2 at v = 0.75, and then decreased to about 8.1 at v = 1.00. For lubricant MCi, the fitted wear rate decreased from approximately 2.1 at v = 0.50 to approximately 0.7 at v = 0.75 and then increased to approximately 3.0 at v = 1.00. These opposing trends account for the very marked Speed × Lubricant interaction.

The interval plots of lubricants and composite materials for COF and ws are shown in Figure 8 and Figure 9.

Residual diagnostics were used to evaluate the adequacy of the mixed-effects models for COF and ws. The residuals were roughly normally distributed, showed no systematic pattern in relation to the fitted values and showed no apparent trend with the order of experimental observation. The results suggest that the assumptions of the fitted mixed-effects model were reasonably met.

## 4. Discussion

### 4.1. Mechanistic Comparison: PA12-CF vs. PA12-GF

Based on the literature reviewed in Section 1.2, the two reinforcement types were expected to produce opposing signatures, with carbon fibers shearing into a low-shear-strength transfer film [7,8] and glass fibers fracturing into abrasive third-body debris [9]. The friction data support that expectation only in part. PA12-GF returned higher friction than PA12-CF under dry sliding, with the difference widening with speed until the two ranges no longer overlapped at 0.75 m/s. It also gave clearly higher friction under L1, where its values occupied the upper part of the range spanned by the carbon fiber composite. Under L2, the two materials were indistinguishable. Differences attributable to material are therefore absent under L2, appreciable under L1, and speed-dependent under dry sliding.

The worn surfaces differed far more consistently. PA12-CF developed an open cavity network in every condition, indicating reinforcement progressively separated from the matrix at the sliding surface, with the severity tracking that of the contact. Under L1, the fibers were exposed as bundles beneath the smeared matrix, while under L2 the cavities were narrower and several retained bodies lighter than the cavity floor, consistent with reinforcement left in place. PA12-GF instead kept a smooth, burnished track throughout, with the reinforcement embedded and level with the matrix. The elemental maps secure this identification, since silicon, aluminum, calcium and oxygen concentrate within the same elongated features while carbon does not. No fiber fracture or third-body fragmentation was found.

Interfacial strength explains this divergence better than reinforcement hardness. Kunishima et al. attributed the inferior behavior of carbon fiber-reinforced PA66 under grease lubrication to insufficient fiber-matrix interfacial strength and found the steel counterface more severely damaged by carbon fiber than by glass fiber composites despite the lower hardness of carbon [10]. Both observations are reproduced here. The cavity network points to reinforcement readily separated from the matrix, whereas the embedded fibers point to the opposite, and iron was quantified above the significance threshold only on PA12-CF under L1 lubrication.

The lubrication condition governs how strongly this material distinction is expressed. Where surface damage is severe, as under L1, the two composites converge in appearance, and the reinforcement contrast recedes behind the damage common to both. Where damage is slight, as under L2, the contrast stands out most clearly. Reinforcement type therefore sets the mode of surface damage, whereas the lubricant determines how far that mode is allowed to develop.

### 4.2. Behavior of the Protic Ionic Liquid Lubricants During Sliding

Although both lubricants belong to the same family of ammonium carboxylate protic ionic liquids, they produced opposite outcomes. MCi lowered the coefficient of friction by roughly an order of magnitude relative to dry sliding and held it stable, while MSu raised it well above the dry baseline with traces that climbed progressively. The same division appears in the thermal record, where MSu produced increases averaging between 5 and 18 °C against less than two degrees for MCi, and in the wear rate, the highest was under MSu and the lowest was under MCi for both composites.

An increase in the viscosity of MSu was observed during testing. Its consequence is documented in Figure 5, where the counterbody carries a consolidated, opaque deposit that has incorporated wear debris, whereas the counterbody from the MCi test retains only thin translucent patches.

For protic ionic liquids of this family, heating is normally expected to reduce viscosity. Kreivaitis et al. report a sharp decrease in the kinematic viscosity of a protic ionic liquid built on the same bis (2-hydroxyethyl) ammonium cation as MSu, from 1271.7 mm^2^/s at 30 °C to 74.2 mm^2^/s at 80 °C, and attribute the lower friction observed at the higher test temperature to the thinner separating film that results [30]. The increase observed here therefore cannot be explained by frictional heating alone and requires a change in the composition of the lubricant itself.

Loss of water offers a plausible explanation, already documented for this compound. Espinosa et al. report that MSu degrades with more than 50% mass loss below 250 °C and attribute a severe friction increase in their own tests to evaporation of water from the sliding interface as the contact temperature rose [13]. Here, the friction under MSu was already elevated within the first minutes, before appreciable heating, so it appears to precede and drive the heating, which would in turn promote water loss, raising viscosity and elevating friction further. Such a self-reinforcing sequence would account both for the progressive rise in the traces and for the concentration of the temperature rise within the first 10 to 20 min. In the case of MCi, the citrate anion is tricarboxylic and carries an additional hydroxyl group against a dicarboxylic succinate [15], which suggests stronger retention of water.

The surface analysis follows the same division, since the lubricant retained on a worn surface was detected only under MSu, through simultaneous enrichment in oxygen and nitrogen. Mass gain rather than loss was also recorded almost exclusively for lubricated tests, with gains reaching several mg, well above the 0.1 mg balance resolution and too large to attribute to wear-driven mass loss, suggesting uptake into the near-surface material, consistent with the known plasticizing action of ionic liquids in polymers [31]. Since surface lubricant was wiped from the disc with isopropyl alcohol before weighing, this gain is unlikely to reflect residual surface film and more plausibly indicates absorption into the near-surface matrix. Ambient humidity can be excluded as the primary source, since the testing atmosphere was controlled and monitored.

### 4.3. Relation Between Worn Surface Topography and Measured Wear Rate

The topography of the worn surfaces does not follow the measured wear rates. PA12-GF retained a smooth and burnished track under every condition, yet its wear rate exceeded that of PA12-CF under dry sliding, 2.81 against 1.83 × 10^−6^ mm^3^/(N·m), and under L2 lubrication, 2.34 against 1.34 × 10^−6^ mm^3^/(N·m). The ranking reverses only under L1, where PA12-CF reached 14.77 against 6.43 × 10^−6^ mm^3^/(N·m) and where its cavity network was also most developed. Appearance alone therefore does not indicate which material lost more volume.

The two quantities describe different aspects of the same process. Wear rate expresses the volume removed from the track, whereas the micrographs describe what remains behind. Material removed uniformly across the contact leaves a smooth track, so a burnished appearance indicates the mode of removal rather than its magnitude. The embedded and level reinforcement observed on PA12-GF is consistent with the matrix and fiber being worn down together, which produces a continuous surface while still consuming material.

For PA12-CF, the discrepancy has a further source. The cavities on those tracks appear to be associated with the reinforcement architecture rather than generated by sliding alone. They were comparable in extent and in individual size under dry and under L2 lubrication, even though the mean wear rate under dry sliding was roughly a third higher than under L2. Their long axes also followed different distributions on different discs. Cavity development is therefore not a proxy for removed volume, and the visually more disrupted appearance of PA12-CF does not imply greater material loss.

### 4.4. Effect of Sliding Speed

Under MCi lubrication, sliding speed produced no consistent effect on friction, with the direction of variation differing between discs of the same condition. Dry sliding separated the two materials, since friction rose from the lowest to the highest speed on every PA12-GF disc, though not monotonically at the intermediate speed on one of them, while PA12-CF showed no common direction.

A clear trend emerged under MSu. For PA12-GF, the coefficient of friction decreased from the lowest to the highest speed on every disc with a complete series, though not monotonically at the highest speed on one of them, falling from about 0.35 at 0.5 m/s to between 0.22 and 0.33 at 1.0 m/s. This finding agrees with the highly significant Material × Speed × Lubricant interaction observed for COF (Table 3, F = 11.24, *p* < 0.001). The pairwise Speed × Lubricant interaction, averaged across materials, was not statistically significant (F = 0.39, *p* = 0.816). However, a speed-dependent trend restricted to a single material–lubricant combination is exactly the behavior that a significant three-way interaction predicts, rather than one that can be detected by a lower-order interaction term. The progressive rise of friction within each test was likewise most pronounced at the lowest speed, as reported in Section 3.1. Both observations point towards a thicker separating film as the entrainment speed increases, which is the direction expected for a viscous lubricant operating in the boundary or mixed regime. The viscosity increase discussed in Section 4.2 would act in the same sense, since a more viscous lubricant reaches a given film thickness at a lower speed. That no equivalent trend appeared under MCi fits a lubricant that remained fluid and produced friction low enough for the regime to be insensitive to speed over the range examined.

The thermal record follows the product of friction and speed rather than speed alone. For PA12-GF, the temperature rise under dry sliding grew steeply with speed, from 1.7 to 12.3 °C, whereas PA12-CF, where friction was low, stayed below 2.5 °C at every speed. Under MSu, the thermal response of PA12-GF peaked at the intermediate speed rather than the highest, reaching 17.6 °C at 0.75 m/s against 9.9 °C at 1.0 m/s. The coefficient of friction meanwhile decreased monotonically over the same range. This pairing supports the shift towards a thicker film described above, since a better separated contact dissipates less energy than heat. The two materials therefore separate thermally only where they also differ in friction. The magnitude of the heating remained modest throughout, with the largest rise recorded in any single test being 20.2 °C above the starting value, so the contact stayed well below the melting and heat deflection temperatures of both filaments.

### 4.5. Disc-to-Disc Variability and Outlier Behavior

The variability recorded between discs tested under identical conditions was modest for most combinations of material, lubrication condition and sliding speed, with a mean relative standard deviation of the coefficient of friction of about 17%. Two situations nevertheless departed from this pattern and merit separate treatment.

The first concerns PA12-CF under MCi lubrication, where the relative standard deviation reached 65 to 67% at the two higher sliding speeds. The absolute values involved are very small, between 0.017 and 0.076, so a difference of a few hundredths translates into a large relative figure. At 0.75 m/s, test CF-L2-08-V2 returned 0.0630 against a group mean of 0.0360. At 1.0 m/s, CF-L2-07-V3 returned 0.0755 and CF-L2-09-V3 returned 0.0174 against a group mean of 0.0439. These deviations occur in both directions and are confined to the condition producing the lowest friction of the study, which suggests intermittent loss and recovery of the lubricant film rather than a systematic difference between specimens.

The second concerns disc GF-D-11 under dry sliding. Its coefficient of friction reached 0.1500 at 1.0 m/s, the highest value under dry sliding and about 44% above the mean of its group. The same track produced a temperature rise of 20.2 °C, matched only by GF-L1-14-V2. Two independently measured quantities therefore depart in the same direction on a single disc, which points to a genuinely different contact rather than an artefact of one measurement. The pre-test roughness of this disc, 0.277 µm, falls within the range of the remaining specimens, so the initial condition of the surface does not account for it. This disc was also the one examined by scanning electron microscopy for the dry condition. Its track appears smooth and burnished, without a cavity network, an appearance consistent with an intense contact accompanied by localized heating and plastic smoothing.

### 4.6. Comparison with the Literature

The agreement with Kunishima et al. regarding damage to the counterface is notable because it holds despite several simultaneous differences [10]. Their work concerns PA66 rather than PA12, injection-molded rather than fused-filament-fabricated specimens, and grease rather than a protic ionic liquid. They nevertheless report what was found here, namely that the carbon fiber composite affects the steel counterface more severely than the glass fiber composite, despite the lower hardness of carbon fiber. In the present study, iron was quantified above the significance threshold only on PA12-CF under L1 lubrication and on no PA12-GF surface. That this outcome persists across such different systems suggests that the fiber–matrix interface, rather than any particular combination of matrix, processing route or lubricant, governs the transfer of counterface material.

Previous work on the lubricated sliding of printed polymers reports friction reduction relative to dry contact [4,5]. The present results show that this cannot be assumed. MSu raised the coefficient of friction well above the dry baseline for both composites and produced the highest wear rates of the study. MCi, by contrast, lowered friction by roughly an order of magnitude. Two lubricants of the same chemical family, applied to the same materials under the same protocol, therefore produced opposite outcomes. Lubricant selection for sliding components made by fused filament fabrication thus requires verification against the specific material rather than an expectation of benefit.

### 4.7. Practical Design Guidance for Sliding-Contact FFF Components

One of the fields tested for PA12-CF under L1 lubrication showed a detectable transfer of iron onto the composite surface, indicating the removal of material from the counterface. Therefore, PA12-GF may be the preferred material when counterface protection is of high importance. This asymmetry is suggestive, but not conclusive, as only one disk per material and condition was examined with SEM/EDX and no quantitative counterface wear measurement was performed for either material. When the polymeric component itself needs to be preserved, PA12-CF showed lower wear rates in dry sliding and under MCi lubrication, as confirmed by profilometric measurement of replicate disks.

Lubricant selection proved more consequential than material selection. The difference between the two lubricants exceeded, by an order of magnitude, the difference between the two composites under any given condition. For a printed sliding component, verifying compatibility with the intended lubricant therefore matters more than optimizing the material.

These findings apply most directly to unlubricated or boundary-lubricated sliding elements such as bushings, guides and low-load bearing surfaces, for which the ball-on-disc configuration used here is a reasonable laboratory analogue. Within that range, the materials were never driven near their thermal limits, so the behavior described above reflects surface processes rather than bulk softening. They extend to lubricated transmission components as well. Polyamide is already established for worm gears and bearing retainers running against steel under grease lubrication [10], and printed nylon gears have been characterized under sliding contact [3].

### 4.8. Limitations

The sliding speed and track radius were confounded by design; each of the three nominal speeds (0.5, 0.75 and 1.0 m/s) was achieved at a fixed, distinct track radius (6.4, 9.6 and 12.7 mm, respectively) on the same disk, so speed and radius could not be independently varied, and any effect attributed to sliding speed cannot be fully separated from a possible effect of radius itself.

Lubricant dosing (20 µL initial, plus periodic top-ups) was identical across all three tracks despite their different circumferences. The contact operates in a boundary or mixed lubrication regime throughout (Section 4.4), in which friction is governed primarily by the adsorbed boundary film rather than by bulk film thickness, so this difference in nominal lubricant volume is expected to have a limited influence on the tribological response.

## 5. Conclusions

This study compared two fused-filament fabricated polyamide 12 composites, one reinforced with short carbon fiber and one with short glass fiber, sliding against a 100Cr6 steel counterface. Testing covered dry conditions and two protic ionic liquid lubricants, at a fixed normal load of 20 N and sliding speeds of 0.5, 0.75 and 1.0 m/s.

The lubricant governed the response more strongly than the material. MCi lowered the mean coefficient of friction to 0.023 for both composites, against a dry mean of 0.071. MSu raised it to a mean of 0.25, an order of magnitude above MCi and a difference exceeding that between the two materials under any given condition.

MSu changed its physical state during testing. The friction traces climbed progressively, the contact-point temperature increases were the largest of the study, and a consolidated deposit incorporating wear debris formed on the counterbody. Oxygen and nitrogen enrichment on the worn surface pointed the same way. MCi remained fluid and produced none of these features.

Both composites ranked the lubrication conditions identically by wear rate, with MSu highest and MCi lowest. Which material wore faster depended on the condition, such as PA12-GF under dry sliding and under MCi, and PA12-CF under MSu, so neither material was superior across the range examined.

Composite material type nevertheless governed the morphology of the worn surface consistently. PA12-CF developed an open cavity network, indicating reinforcement progressively separated from the matrix. PA12-GF retained a smooth, burnished track with the reinforcement embedded and level, an identification secured by the co-localization of silicon, aluminum and calcium. Surface appearance did not predict wear rate, since the smoother material was not consistently the one that lost less volume. Iron transfer to the composite surface, discussed further below, reproduces a result previously reported for a system differing in matrix, processing route and lubricant.

Frictional heating remained modest throughout, with the largest rise recorded in any single test being 20.2 °C above the starting temperature, which places the observed behavior firmly in the domain of surface processes. The variability between nominally identical specimens was modest for most conditions, although individual discs differed appreciably from their replicates, which justified treating the disc as the independent replicate unit.

PA12-CF showed lower wear rates than PA12-GF under dry sliding and MCi lubrication, confirmed profilometrically across replicate discs. Iron transfer to the composite surface was detected in one examined field for PA12-CF and none for PA12-GF; given the limited SEM sampling and the absence of any quantitative counterface wear measurement, this is a qualitative observation warranting further investigation rather than a confirmed protective effect. The two composites therefore represent a trade-off rather than a ranking, with lubricant compatibility taking precedence over the material.

## Figures and Tables

**Table 1:** Key mechanical and thermal properties of PA12-CF and PA12-GF filaments [18,19].

Property	PA12-CF	PA12-GF
Reinforcement content	20 wt% carbon fiber	15 wt% glass fiber
Density	1.08 g/cm^3^	1.13 g/cm^3^
Tensile strength	86 ± 6 MPa	72 MPa
Modulus	5800 ± 300 MPa (flexural) ^1^	3100 MPa (tensile) ^1^
Elongation at break	13.7 ± 2.5%	8%
Notched impact strength	11.3 ± 2.5 kJ/m^2^	16 kJ/m^2^
Heat deflection temperature (0.45 MPa)	175 ± 3 °C	175 °C
Glass transition temperature	99 °C	not reported
Melting temperature	190 °C	not reported
Filament diameter	1.75 mm	1.75 mm

Modulus values are reported by the two manufacturers under different test modes (flexural for PA12-CF, tensile for PA12-GF) and are not directly comparable.

**Table 2:** Coefficient of friction, pre-test surface roughness (Ra), profilometric specific wear rate, and contact-point temperature rise (ΔT) for all individual tests.

Test	v [m/s]	Ra (µm)	COF	COF Mean ± SD	Δm (g)	ΔT (°C)	ΔT Mean ± SD	ws Prof. (×10^−6^ mm^3^/N·m)	ws Mean ± SD
CF-D-01-V1	0.5	0.256	0.0575	0.0659 ± 0.0074	0.0002	0.4	0.9 ± 0.5	1.281	1.55 ± 0.39
CF-D-02-V1	0.5	0.247	0.0684		0.0005	0.9		1.385	
CF-D-03-V1	0.5	0.289	0.0717		0.0002	1.3		1.994	
CF-D-01-V2	0.75	0.256	0.0694	0.0637 ± 0.0052	0.0004	1.6	1.3 ± 0.3	1.401	1.67 ± 0.24
CF-D-02-V2	0.75	0.247	0.0593		0.0001	1.0		1.835	
CF-D-03-V2	0.75	0.289	0.0624		0.0002	1.2		1.788	
CF-D-01-V3	1	0.256	0.0751	0.0624 ± 0.0120	0.0006	2.8	2.4 ± 0.7	2.610	3.18 ± 0.54
CF-D-02-V3	1	0.247	0.0608		−0.0007	1.6		3.692	
CF-D-03-V3	1	0.289	0.0513		0.0000	2.7		3.249	
CF-L1-04-V1	0.5	0.242	0.12	0.1587 ± 0.0417	0.0006	12.2	5.3 ± 6.0	6.921	7.52 ± 0.56
CF-L1-05-V1	0.5	0.298	0.1532		−0.0002	2.1		8.029	
CF-L1-06-V1	0.5	0.263	0.2029		0.0010	1.7		7.611	
CF-L1-04-V2	0.75	0.242	0.2378	0.2320 ± 0.0211	0.0001	10.6	5.6 ± 4.3	15.080	15.10 ± 0.17
CF-L1-05-V2	0.75	0.298	0.2087		0.0004	3.0		15.280	
CF-L1-06-V2	0.75	0.263	0.2496		−0.0013	3.3		14.950	
CF-L1-04-V3	1	0.242	0.1973	0.2372 ± 0.0477	0.0009	12.3	9.0 ± 3.7	14.77	14.48 ± 1.19
CF-L1-05-V3	1	0.298	0.2244		0.0018	9.6		15.490	
CF-L1-06-V3	1	0.263	0.29		0.0018	5.0		13.170	
CF-L2-07-V1	0.5	0.256	0.0189	0.0211 ± 0.0021	0.0003	1.4	1.2 ± 0.2	2.14	1.58 ± 0.49
CF-L2-08-V1	0.5	0.267	0.023		0.0003	1.0		1.26	
CF-L2-09-V1	0.5	0.257	0.0215		−0.0005	1.1		1.344	
CF-L2-07-V2	0.75	0.256	0.0225	0.0360 ± 0.0234	0.0000	1.6	1.4 ± 0.4	0.862	0.59 ± 0.24
CF-L2-08-V2	0.75	0.267	0.0630		0.0003	1.6		0.511	
CF-L2-09-V2	0.75	0.257	0.0224		0.0002	0.9		0.404	
CF-L2-07-V3	1	0.256	0.0755	0.0439 ± 0.0294	−0.0009	2.5	2.0 ± 0.6	2.207	1.91 ± 0.27
CF-L2-08-V3	1	0.267	0.0389		−0.0006	1.4		1.857	
CF-L2-09-V3	1	0.257	0.0174		0.0002	2.0		1.666	
GF-D-10-V1	0.5	0.260	0.0812	0.0709 ± 0.0140	0.0003	3.1	1.7 ± 1.2	2.390	2.94 ± 0.63
GF-D-11-V1	0.5	0.277	0.0766		0.0000	0.9		2.815	
GF-D-12-V1	0.5	0.255	0.0550		0.0009	1.2		3.628	
GF-D-10-V2	0.75	0.260	0.0846	0.0830 ± 0.0083	0.0004	9.1	7.0 ± 2.9	4.761	4.80 ± 0.13
GF-D-11-V2	0.75	0.277	0.0904		0.0022	8.1		4.950	
GF-D-12-V2	0.75	0.255	0.0741		0.0005	3.7		4.698	
GF-D-10-V3	1	0.260	0.091	0.1040 ± 0.0411	0.0020	13.5	12.3 ± 8.6	2.56	2.36 ± 0.21
GF-D-11-V3	1	0.277	0.15		0.0023	20.2		2.387	
GF-D-12-V3	1	0.255	0.0709		0.0017	3.2		2.144	
GF-L1-13-V1	0.5	0.274	0.3522	0.3494 ± 0.0033	0.0001	6.5	9.0 ± 2.4	7.070	6.86 ± 0.60
GF-L1-14-V1	0.5	0.247	0.3503		0.0016	9.3		7.328	
GF-L1-15-V1	0.5	0.274	0.3457		0.0023	11.2		6.187	
GF-L1-13-V2	0.75	0.274	0.3039	0.2797 ± 0.0300	0.0030	15.1	17.6 ± 2.6	7.470	7.33 ± 0.84
GF-L1-14-V2	0.75	0.247	0.2892		0.0004	20.2		6.431	
GF-L1-15-V2	0.75	0.274	0.2461		0.0021	17.4		8.097	
GF-L1-13-V3	1	0.274	0.33	0.2652 ± 0.0576	0.0042	9.2	9.9 ± 0.8	2.11	1.49 ± 0.54
GF-L1-14-V3	1	0.247	0.2455		0.0037	9.8		1.216	
GF-L1-15-V3	1	0.274	0.22		0.0035	10.8		1.137	
GF-L2-16-V1	0.5	0.252	0.0198	0.0211 ± 0.0022	0.0003	0.0	0.6 ± 0.6	3.314	2.64 ± 0.58
GF-L2-17-V1	0.5	0.272	0.0199		−0.0002	0.6		2.275	
GF-L2-18-V1	0.5	0.252	0.0237		0.0006	1.2		2.344	
GF-L2-16-V2	0.75	0.252	0.0202	0.0233 ± 0.0034	−0.0004	1.1	1.5 ± 0.5	1.054	0.83 ± 0.26
GF-L2-17-V2	0.75	0.272	0.0226		−0.0005	1.3		0.548	
GF-L2-18-V2	0.75	0.252	0.027		0.0005	2.1		0.899	
GF-L2-16-V3	1	0.252	0.0234	0.0230 ± 0.0071	0.0003	0.8	1.2 ± 0.5	4.577	4.08 ± 0.64
GF-L2-17-V3	1	0.272	0.0157		−0.0006	1.7		3.353	
GF-L2-18-V3	1	0.252	0.0298		0.0016	1.0		4.299	

* Test codes follow the format [Material]−[Condition]−[Disc]−[sliding speed], where Material is CF (PA12-CF) or GF (PA12-GF), Condition is D (dry), L1 (MSu lubricant), or L2 (MCi lubricant), and the final label (V1, V2, V3) denotes the nominal sliding speed tested of V1 = 0.50 m/s, V2 = 0.75 m/s, and V3 = 1.00 m/s, obtained by testing at a fixed track radius (6.4, 9.6 and 12.7 mm respectively). Mean ± standard deviation values are calculated across the three replicate discs. ΔT is the contact-point temperature rise, measured as the difference between the 60 min and the baseline recorded immediately before the start of the test.

**Table 3:** Mixed-effects analysis on mean steady-state COF.

Source		COF			
	DF Num	DF Den	F-Value	*p*-Value	PC
Material	1.00	12.00	11.85	0.005	S
v	2.00	24.00	0.75	0.484	I
Lubricant	2.00	12.00	203.43	<0.001	HS
Material*v	2.00	24.00	8.65	0.001	S
Material*Lubricant	2.00	12.00	9.31	0.004	S
v*Lubricant	4.00	24.00	0.39	0.816	I
Material*v*Lubricant	4.00	24.00	11.24	<0.001	HS
	R_sq = 98.13%				

Note: HS—highly significant; S—significant; I—insignificant; * indicates interaction between factors.

**Table 4:** Mixed-effects analysis on specific wear rate, ws.

Term		ws			
	DF Num	DF Den	F-Value	*p*-Value	PC
Material	1.00	12.00	85.13	<0.001	S
v	2.00	24.00	27.80	<0.001	S
Lubricant	2.00	12.00	635.60	<0.001	HS
Material*v	2.00	24.00	94.68	<0.001	S
Material*Lubricant	2.00	12.00	262.17	<0.001	HS
v*Lubricant	4.00	24.00	62.95	<0.001	S
Material*v*Lubricant	4.00	24.00	88.15	<0.001	S
	R_sq = 98.22%				

Note: HS—highly significant, S—significant; * indicates interaction between factors.

## Data Availability

Original contributions presented in this study are included in this article; further inquiries can be directed to the corresponding authors.

## Abbreviations

The following abbreviations are used in this manuscript:

FFF	fused filament fabrication
PA12-CF	carbon fiber-reinforced polyamide 12
PA12-GF	glass fiber-reinforced polyamide 12
CoF	coefficient of friction
ABS	acrylonitrile butadiene styrene
PC	polycarbonate
PLA	polylactic acid
IL	Ionic liquid
PIL	protic ionic liquid
MSu	bis(2-hydroxyethyl)ammonium succinate
MCi	bis(2-hydroxyethyl)ammonium citrate
SLS	selective laser sintering
PA66	polyamide 66
WTA	wear track area
ws	specific wear rate
SEM	scanning electron microscopy
EDX	energy dispersive X-ray spectroscopy
SD	standard deviation
